# Dual PDF Signaling Pathways Reset Clocks Via TIMELESS and Acutely Excite Target Neurons to Control Circadian Behavior

**DOI:** 10.1371/journal.pbio.1001810

**Published:** 2014-03-18

**Authors:** Adam Seluzicki, Matthieu Flourakis, Elzbieta Kula-Eversole, Luoying Zhang, Valerie Kilman, Ravi Allada

**Affiliations:** Department of Neurobiology, Northwestern University, Evanston, Illinois, United States of America; New York University, United States of America

## Abstract

Molecular circadian clocks are interconnected via neural networks. In *Drosophila*, PIGMENT-DISPERSING FACTOR (PDF) acts as a master network regulator with dual functions in synchronizing molecular oscillations between disparate PDF(+) and PDF(−) circadian pacemaker neurons and controlling pacemaker neuron output. Yet the mechanisms by which PDF functions are not clear. We demonstrate that genetic inhibition of protein kinase A (PKA) in PDF(−) clock neurons can phenocopy PDF mutants while activated PKA can partially rescue PDF receptor mutants. PKA subunit transcripts are also under clock control in non-PDF DN1p neurons. To address the core clock target of PDF, we rescued *per* in PDF neurons of arrhythmic *per^01^* mutants. PDF neuron rescue induced high amplitude rhythms in the clock component TIMELESS (TIM) in *per*-less DN1p neurons. Complete loss of PDF or PKA inhibition also results in reduced TIM levels in non-PDF neurons of *per^01^* flies. To address how PDF impacts pacemaker neuron output, we focally applied PDF to DN1p neurons and found that it acutely depolarizes and increases firing rates of DN1p neurons. Surprisingly, these effects are reduced in the presence of an adenylate cyclase inhibitor, yet persist in the presence of PKA inhibition. We have provided evidence for a signaling mechanism (PKA) and a molecular target (TIM) by which PDF resets and synchronizes clocks and demonstrates an acute direct excitatory effect of PDF on target neurons to control neuronal output. The identification of TIM as a target of PDF signaling suggests it is a multimodal integrator of cell autonomous clock, environmental light, and neural network signaling. Moreover, these data reveal a bifurcation of PKA-dependent clock effects and PKA-independent output effects. Taken together, our results provide a molecular and cellular basis for the dual functions of PDF in clock resetting and pacemaker output.

## Abstract 2

Studies in *Drosophila* circadian neurons reveal a bifurcation in the Pigment Dispersing Factor (PDF) neuropeptide signaling pathway, independently synchronizing circadian clocks via PKA or acutely controlling neuronal excitability via cAMP.

## Author Summary

Circadian clocks provide a mechanism for predicting and adapting behavioral and physiological processes to 24-hour rhythms in the environment. In animal nervous systems, cell-autonomous molecular oscillators are coupled via neural networks that control daily patterns of activity. A major neuropeptide synchronizing neural oscillators in the *Drosophila* clock network is PIGMENT DISPERSING FACTOR (PDF). Here we identify a fork in the processing of the PDF signal in circadian neurons to independently reset the molecular clock and regulate neuronal activity. We show that the cAMP-activated protein kinase A (PKA) in circadian neurons is necessary and sufficient for many PDF-dependent behaviors. In addition, we find that a PDF>PDF receptor>PKA pathway targets the clock component TIMELESS to control molecular oscillators, and that this process may be influenced by rhythmic expression of PKA. We show that this pathway splits at the level of cAMP generation, with PDF and cAMP acutely increasing the activity of clock neurons in a PKA-independent manner. Thus, PDF operates via dual signaling pathways: one via PKA to reset clocks and the other via cAMP to acutely control activity. These results have broad implications given the conserved involvement of neuropeptide signaling in synchronizing clocks in circadian neural networks.

## Introduction

Circadian clocks endow organisms with the ability to predict and respond adaptively to daily changes in the environment. In many taxa, these clocks consist of cell-autonomous molecular feedback loops, producing ∼24-hour oscillations at the mRNA and protein levels. In insects and mammals these clocks are also connected in neural networks that stabilize and synchronize these molecular feedback loops and communicate timing information to regulate daily behavior. How network and cell-autonomous mechanisms collaborate to produce robust circadian rhythms remains a major question.

In *Drosophila*, the molecular circadian clock consists of a set of interlocked transcriptional feedback loops in which the basic helix-loop-helix *per-arnt-sim* (bHLH-PAS) domain transcription factor CLOCK (CLK) forms a heterodimer with CYCLE (CYC) and binds E-boxes in the promoter regions of *period* (*per*), *timeless* (*tim*), *vrille* (*vri*), *Par-domain protein 1ε* (*Pdp1ε*) and *clockwork orange* (*cwo*), promoting their transcription (reviewed in [1]). PDP1ε and VRI feed back to regulate the *Clk* and *cryptochrome* (*cry*) promoters [2],[3], while CWO feeds back to regulate CLK/CYC activation at E-boxes [4]–[7]. PER and TIM proteins dimerize in the cytosol and are each required for their subsequent localization to the nucleus where PER inhibits CLK/CYC–mediated activation [8]–[14]. The CRY photoreceptor mediates light resetting via TIM degradation [15]–[18]. Clock function is evident as 24-h oscillations in the mRNA and protein levels of most of these clock components. The activity, stability, and subcellular localization of these proteins are largely controlled post-translationally by daily phosphorylation rhythms and subsequently by ubiquitin/proteasome dependent degradation [17],[19]–[25]. In contrast to transcriptional regulators, significant oscillations have not been described for these post-translational regulators with the exception of the PP2A subunits *tws* and *wdb* [26].

In insects and mammals, intercellular signaling among pacemaker neurons in neural networks has been found to be critical for synchronizing molecular clocks. The *Drosophila* pacemaker network is comprised of ∼150 neurons of which specific subgroups regulate discrete aspects of behavior in light-dark (LD) and constant darkness conditions (DD) [27]. Two of these groups—all but one sLNv and all lLNvs (small and large ventral-lateral neurons) express the neuropeptide PIGMENT DISPERSING FACTOR (PDF). The s-LNvs rhythmically express PDF in the dorsally projecting terminals that terminate near the DN1 [28]. Loss of function of either *pdf* or its receptor *pdfr* results in strongly reduced morning anticipation, an evening activity peak that is phase-advanced by 1 h relative to wild-type, and strongly reduced DD rhythmicity [29]–[41]. Ablation of PDF neurons results in similar phenotypes suggesting that PDF is the major transmitter of these neurons [37]. Transgenic rescue of *pdfr* mutants showed morning anticipation could be attributed to function in the DN1p neurons, while evening anticipation phenotypes mapped to non-PDF neurons, including the PDF(−) sLNv, the CRY+ subset of the LNd, and the DN1 [35],[41].

PDF coordinates molecular oscillations between disparate circadian pacemaker neurons and mediates pacemaker neuron output downstream of the clock. *tim* and *cry* mRNA oscillations in pacemaker neurons are damped in *pdf^01^* mutants [42]. The timing of nuclear entry of PER protein in sLNv becomes phase-dispersed in DD in *pdf^01^* flies [43]. PER expression in the LNd and DN1 cells is phase advanced on the first day of DD and subsequently damps [34],[43]. Analysis of TIM protein levels and a PER-luciferase fusion reporter suggested that the clocks in the different cell groups in the network can both advance or delay in response to PDF signaling [44].

Interestingly, while *pdfr* mutants exhibit notably reduced morning anticipation, PER oscillations in sLNvs and DN1s are comparable to wild-type flies under LD conditions [35], suggesting that PDF also mediates pacemaker neuron output in clock target neurons. Acute activation and silencing of neuronal activity observed by PDF injection in cockroaches are consistent with this latter mechanism [45]. However, the underlying signaling pathways mediating these dual PDF functions within non-PDF clock neurons, i.e., clock resetting and neural output, have not been identified.

## Results

### Reducing Protein Kinase A Activity in Non-PDF Circadian Neurons Mimics Loss of PDF/PDFR

PDFR is expressed in most of the pacemaker neurons and most of these neurons respond to PDF application in *ex vivo* preparations with increases in cAMP levels [46],[47]. We tested the function of the cAMP-dependent protein kinase A (PKA) in clock neuron-driven behavior. cAMP is the canonical activator of PKA activity. cAMP binds the PKA regulatory (R) subunit releasing the catalytic (C) subunit to phosphorylate substrates (reviewed in [48]). While PKA signaling has been implicated in circadian clock function [49],[50], its precise role in mediating PDFR signaling has not been defined.

**Figure 1:**
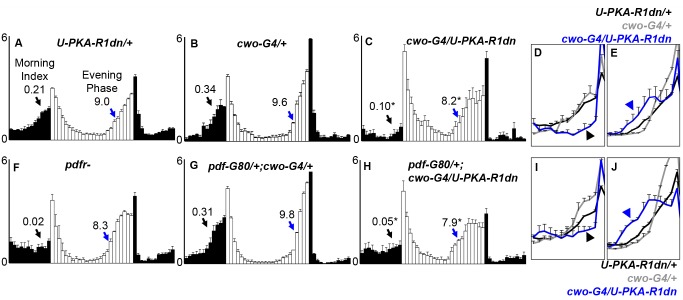
Expression of a dominant-negative PKA regulatory subunit (PKA-R1dn) can phenocopy *pdfr*- behavior phenotypes. Histograms are normalized activity profiles of flies in 12;12 h dark (LD). Black bars indicate activity occurring in the dark phase, white bars indicate activity occurring in the light phase. Error bars are SEM. Genotype (N). (A) *U-PKA-R1dn*/+ (74), (B) *cwo-G4*/+ (16), (C) *cwo-G4/U-PKA-R1dn* (16), (F) *pdfr-* (32), (G) *pdfG80*/+;*cwo-G4*/+ (45), (H) *pdf-G80*/+;*cwo-G4*/*U-PKA-R1dn* (32). Blue arrowheads and associated values (onset time in ZT ± SEM) indicate the onset of evening activity as described [35], with the onset time defined as the first point in the largest increase in activity calculated over a 2-h (four 30-min bins) sliding window. Black arrowheads and associated values (morning index ± SEM) indicate morning anticipation. Morning index was calculated using a variant of the method described in [32] (See Materials and Methods for details on behavior quantification methods). (D,E,I,J) magnified overlays of morning (D, I) and evening (E, J) for the indicated color-coded genotypes. Graphs show the 6 h prior to the D>L (morning) or L>D (evening) transitions, as well as the first bin of the L or D phase, respectively. *p<0.05 versus both G4 and U parental controls.

Using the Gal4-UAS system, we expressed a type I regulatory subunit of PKA that is defective for cAMP binding (*U-PKA-R1dn*), thereby rendering endogenous catalytic subunits insensitive to cAMP activation [51],[52]. Initially we drove expression using circadian drivers, such as *cwo-G4* (also known as c632a; [5]) and examined behavior under standard 12:12LD conditions. *cwo-G4* is a GAL4 enhancer trap insertion just upstream of the transcription start site of core clock gene *cwo* and drives expression in clock neurons [5]. In addition to expression in the PDF clock neurons [5], we examined *cwo-G4* driven nuclear green fluorescent protein (GFP) expression in pacemaker neurons using PER co-labeling. We observed GFP expression mainly in the circadian pacemaker neurons, with limited expression in other brain regions (Figure S1; unpublished data). Among pacemaker neurons, we observed GFP in non-PDF clock neurons, including all of the LNds and the PDF(−) s-LNv, as well as a number of DN1s and DN3s (Figure S1). Broad expression of PKA-R1dn with *cwo-G4* (Figure 1C) was able to phenocopy many features seen in *pdfr* mutants (Figure 1D). These flies exhibited reduced morning anticipation and phase advanced evening activity under LD cycles, and were nearly arrhythmic in constant darkness, thus mimicking the three canonical behavioral phenotypes of *pdf* and *pdfr* mutants (Figure 1A–1E; Tables 1 and 2).

**Table 1:** LD morning anticipation and evening phase measures.

Genotype	Morning Index ± SEM	Evening Activity Onset ZT ± SEM	*n*
***pdfr-***	0.02±0.03	8.3±0.1	32
***U-PKA-R1dn/+***	0.21±0.01	9.0±0.1	74
***cwo-G4/+***	0.34±0.02	9.6±0.2	16
***cwo-G4/U-PKA-R1dn***	0.10±0.04*	8.2±0.3*	16
***pdf-G80/+;cwo-G4/+***	0.31±0.02	9.8±0.1	45
***pdf-G80/+;cwo-G4/U-PKA-R1dn***	0.05±0.04*	7.9±0.2*	34
***pdf-G80/+;cry13-G4/+***	0.12±0.02	9.8±0.2	22
***pdf-G80/+;cry13-G4/U-PKA-R1dn***	0.07±0.02^a^	8.0±0.2*	10
***pdfr-;;U-PKA-R1dn/+***	0.06±0.02	7.3±0.1	49
***pdfr-;pdf-G80/+;cry13-G4/U-PKA-R1dn***	0.06±0.03	8.1±0.2	26
***Clk4.1-G4/+***	0.21±0.03	9.4±0.1	19
***Clk4.1-G4/U-PKA-R1dn***	0.12±0.03*	9.0±0.2	14
***mai179-G4/+;pdf-G80/+***	0.11±0.01	9.7±0.2	31
***mai179-G4/+;pdf-G80/U-PKA-R1dn***	0.18±0.02	8.2±0.2*	26
*pdf-G80/U-mC* * *;cry13-G4/+*	0.18±0.02	9.1±0.3	15
***pdfr-;U-mC*** * ***/+***	0.03±0.02	8.0±0.2	22
*pdfr-;pdf-G80/U-mC* * *;cry13-G4/+*	0.14±0.01^b^	9.2±0.1^b^	48

See Materials and Methods for details of behavior quantification.

a *p* = 1.01×10−5 versus *U-PKA-R1dn*/+, *p* = 0.056 versus *pdf-G80*/+;*cry13-G4*/+.

b ns versus pdf-G80/U-PKA-mC*;cry13-G4/+.

*p≤0.025 versus both parental controls.

**Table 2:** DD circadian behavior parameters.

Genotype	Period ± SEM	P-S ± SEM	*n*	%R
***pdfr-***	23.7±0.3	7±2	32	19
***U-PKA-R1dn/+***	23.5±0.0	92±5	63	100
***cwo-G4/+***	23.7±0.1	86±11	16	100
***cwo-G4/U-PKA-R1dn***	24.0±1.1	7±3*	16	25
***pdf-G80/+;cwo-G4/+***	23.6±0.1	45±4	45	93
***pdf-G80/+;cwo-G4/U-PKA-R1dn***	23.6±0.1	29±5*	32	66
***pdf-G80/+;cry13-G4/+***	23.9±0.1	117±10	14	100
***pdf-G80/+;cry13-G4/U-PKA-R1dn***	23.8±0.1	50±12*	10	100
***pdfr-;;U-PKA-R1dn/+***	23.3±0.2	5±1	48	25
***pdfr-;pdf-G80/+;cry13-G4/U-PKA-R1dn***	21.5^a^	1±1	26	4
***Clk4.1-G4/+***	23.9±0.1	120±9	19	100
***Clk4.1-G4/U-PKA-R1dn***	24.1±0.2	38±7*	13	92
***mai179-G4/+;pdf-G80/+***	23.8±0.1	84±8	30	93
***mai179-G4/+;pdf-G80/U-PKA-R1dn***	23.6±0.1	69±7	26	96
***pdfr-;U-PKA-mC*** * ***/+***	23.6±0.2	11±3	22	41
***U-PKA-mC*** * ***/+;cry13-G4/+***	24.0±0.1	37±6	23	78
***pdfr-;u-PKA-mC*** * ***/+;cry13-G4/+***	24.7±0.3	6±2	30	17
***pdfr-;pdf-G80/U-PKA-mC*** * ***;cry13-G4/+***	24.8±0.6	3±2	43	7
***pdf-G80/U-PKA-mC*** * ***;cry13-G4/+***	23.9±0.1	43±7	14	100

See Materials and Methods for details of behavior quantification.

a Only one rhythmic fly, therefore there is no SEM.

*p<0.014 versus both parental controls.

Given that the majority of PDFR functions mapped to non-PDF clock neurons, we then asked whether these PKA-R1dn effects were due to its expression in non-PDF neurons by blocking PDF neuron expression with *pdf-G80*. Importantly, we did not detect *cwo-G4* driven GFP expression in the PDF(+) neurons, verifying effective inhibition of *cwo-G4* activity in those neurons by *pdf*-*G80* (Figure S1). We observed once again that these flies behaved similarly to *pdf* and *pdfr* mutants, exhibiting the three hallmark phenotypes of reduced morning anticipation, phase-advanced evening activity, and reduced rhythmicity in constant darkness (Figure 1F–1J; Table 1), providing strong evidence for PKA function in non-PDF neurons in mediating PDF effects in circadian neurons. The addition of *pdf-G8*0 did improve the rhythmicity (power-significance [P-S]) in constant darkness, suggesting that PKA activity in the PDF neurons contributes to a portion of this phenotype (Table 2, *p*<0.005). Nonetheless, the morning and evening phenotypes continue to map to non-PDF neurons.

**Figure 2:**
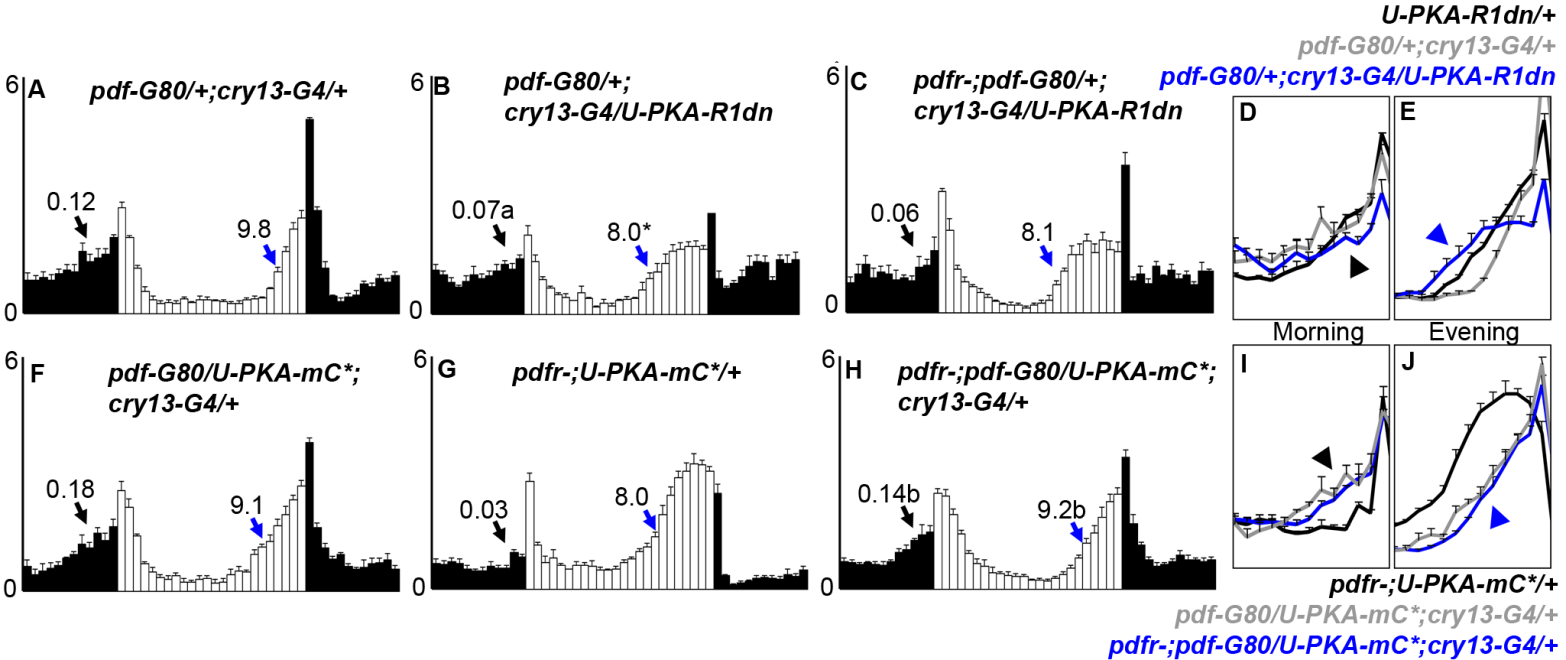
Non-PDF (E-cell) pacemaker neuron specific modulation of PKA activity can phenocopy or rescue *pdfr*- behaviors. E-cells were targeted using *cry13-G4* and *pdf-G80*. U-PKA-mC* is a constitutively active PKA catalytic subunit that lacks the ability to bind to regulatory subunits. Graphs are as in Figure 1. Genotype (N). (A) pdf-G80/+;cry13-G4/+ (15), (B) pdf-G80/+;cry13-G4/U-PKA-R1dn (10), (C) pdfr-;pdf-G80/+;cry13-G4/U-PKA-R1dn (26), (F) pdf-G80/U-PKA-mC*;cry13-G4/+ (15). (G) pdfr-;U-PKA-mC*/+ (22), (H) pdfr-;pdf-G80/U-PKA-mC*;cry13-G4/+ (48). (D,E,I,J) magnified overlays of morning (D,I) and evening (E,J) for the indicated color-coded genotypes as in Figure 1. *p<0.05 versus both G4 and UAS parental controls. a, *p*<0.05 versus UAS parental control, *p* = 0.056 versus G4 parental control. b, *p*<0.05 versus *pdfr*-;U-PKA-mC*/+, not significant versus *pdf-G80*/U-PKA-mC*;*cry13-G4*/+. Complete quantification appears in Table 1.

Given that *cwo-G4* also drives limited expression in some non-circadian areas we decided to examine PKA function using an independent circadian driver. Previous work from our laboratory demonstrated that morning and evening anticipation phenotypes of *pdfr* mutant behavior can be rescued by expressing *U-pdfr* in non-PDF neurons using *cry13-G4* and *pdf-G80* [35]. Here we expressed *U-PKA-R1dn* in PDF(−) circadian cells using this Gal4/Gal80 combination and found that inhibition of PKA activity also results in morning and evening anticipation similar to *pdfr-* (Figure 2A, 2B, 2D, 2E; Table 1). If PDFR and PKA are operating in the same pathway, we would expect that *U-PKA-R1dn* expression in a *pdfr^han5304^ (pdfr-)* mutant background would exhibit phenotypes comparable to either PKA-R1dn or *pdfr* mutants alone. Indeed, we found no differences in morning or evening behaviors among *pdfr-*, PKA-R1dn expression, or PKA-R1dn expression in the *pdfr-* background using *cry13-G4/pdf-G80* (Figure 2B, 2C; Table 1), suggesting that PDFR and PKA operate in a common pathway within these cells.

### Expression of a Constitutively Active PKA Can Partially Rescue PDFR Mutant Phenotypes

Rescue or suppression of mutant receptor function by expression of activated downstream signaling components is a powerful *in vivo* method to elucidate signaling pathways [53]. We also attempted to suppress *pdfr* mutant phenotypes by expressing a PKA catalytic subunit (U-PKA-mC*), *which is* defective for regulatory subunit binding and is therefore constitutively active [52]. We expressed PKA-mC* in PDF(−) circadian neurons in *pdfr* mutants and observed that it rescued the reduced morning anticipation and phase-advanced evening activity onset characteristic of *pdfr* mutants (Figure 2; Table 1). There were no notable effects of PKA-mC* expression in a wild-type background in LD (Figure 2F–2J; Table 1). While PKA-mC* rescued LD phenotypes, we did not observe rescue of DD rhythms, suggesting that PKA-mC* is not expressed at the appropriate levels, cAMP inducibility may be required, and/or that non-PKA signaling pathways may contribute to PDFR function in DD (Table 2). We obtained similar results in the absence of *pdf-G80*, thus allowing PKA-mC* expression in the PDF neurons, suggesting that lack of PKA activity in PDF neurons is not responsible for the lack of DD rhythm rescue by PKA-mC* (Table 2). Taken together, our genetic evidence, especially our ability to bypass PDFR function with an activated form of PKA, indicate that PKA is a major mediator of PDFR signaling in non-PDF clock neurons. PKA activity is both necessary and sufficient for the execution of most PDFR-mediated behaviors.

### Circadian PKA Function and Expression in DN1p Neurons

**Figure 3:**
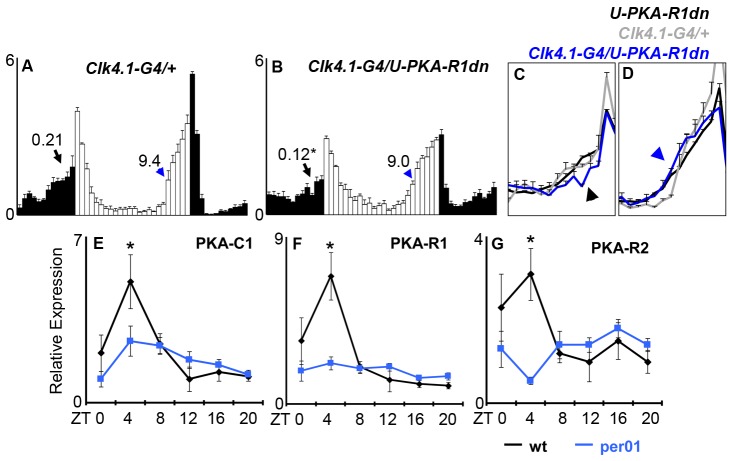
PKA function in DN1p neurons. (A) Reduction of PKA activity in DN1p has no effect on the phase of evening activity, but reduces morning anticipation. Graphs are as in Figure 1. Genotype (N). (A) Clk4.1-G4/+ (19), (B) Clk4.1-G4/U-PKA-R1dn (14). (C and D) are overlays of morning and evening activity as in Figure 1. PKA subunits are transcriptionally regulated by the circadian clock in DN1ps. DN1ps were marked with *Clk4.1-G4*>*U-GFP* in wt (black) or *per^01^* (blue) background, dissociated, sorted, and analyzed by quantitative RT-PCR for PKA subunit transcripts (see Materials and Methods). (E) PKA-C1, (F) PKA-R1, (G) PKA-R2. Error bars are SEM.

We have previously shown that we can rescue *pdfr* mutant morning anticipation and DD rhythmicity, but not evening anticipation, by expressing wild-type *pdfr* only in DN1p circadian neurons using the *Clk4.1-G4* driver [41]. To test whether PKA also functions in the DN1p we expressed PKA-R1dn using *Clk4.1-G4* and found modestly reduced morning anticipation and DD rhythmicity, but no change in evening anticipation, complementing our observations for *pdfr* rescue (Figure 3A–3D; Tables 1 and 2) [35],[41]. Given that *cwo-G4/pdf-G80* driven PKA-RIdn exhibited more robust morning and DD phenotypes (Tables 1 and 2), we hypothesize that non-PDF, non-DN1p cells may also contribute to these phenotypes and/or this driver combination more strongly inhibits PKA within the DN1p than *Clk4.1-G4*. To address the neural substrates of PKA function in evening anticipation, we used the *mai179-G4* driver in combination with *pdf-G80*, which drives expression in the single PDF(−) sLNv, and the CRY(+) subset of LNd with variable expression in a 1–2 DN1s [30],[54],[55]. Here we found that PKA inhibition phase advances evening anticipation similarly to *pdfr* mutants (Figure S2). In keeping with a model in which the CRY+ LNd and fifth PDF(−) sLNv selectively regulate evening anticipation, PKA-R1dn driven by *mai179-G4*/*pdf-G80* had no effect on morning anticipation (Figure S2; Table 1) or DD rhythms (Table 2).

Rhythms in PDF levels are apparent in the terminals of LNv neurons and rhythmic PDF release is thought to contribute to the temporal encoding of the PDF signal; however, rhythmic PDF may not be necessary for rhythmic behavior or clock function [28],[40],[56]. We hypothesized that rhythmic control of signal processing within cells receiving the PDF signal may contribute to the robustness of this pathway. To determine whether PKA subunit transcripts are under circadian clock control in DN1p clock neurons, we expressed UAS-membrane GFP (*U-mGFP*) using *Clk4.1-G4* and isolated these neurons by fluorescence-activated cell sorting (FACS). After RNA isolation and linear amplification (see Materials and Methods), we examined the transcript levels of the three catalytic PKA subunits (C1, C2, and C3) and two regulatory subunits (R1 and R2) by quantitative real-time (RT)-PCR. Transcript levels of three PKA subunits (PKA-C1, PKA-R1, and PKA-R2) oscillate in phase, with coincident peaks in the mid-day (ZT4) (Figure 3E–3G). Peak transcript levels were reduced and rhythms were not detected in the *per^01^* mutants consistent with circadian clock control. The two other PKA transcripts (C2, C3) were near the limits of quantitative detection. Thus, not only is PKA activity likely controlled via rhythmic inputs of PDF-driven cAMP production but PKA is also rhythmically controlled at the level of gene expression. We hypothesize that these dual mechanisms collaborate to provide a robust time-of-day PKA signal to synchronize non-PDF to PDF oscillators.

### PDF-Neurons Specifically Control TIM Protein Levels in Non-PDF Circadian Neurons

**Figure 4:**
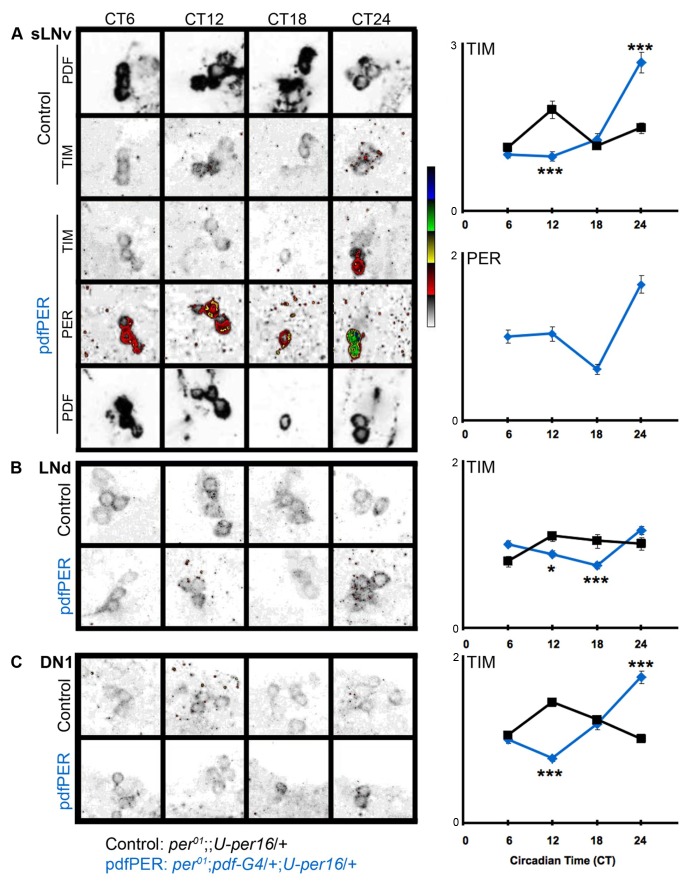
PDF cell clocks specifically target TIM in LNds and DN1s in constant darkness. Single confocal slices showing PDF, PER, and TIM staining. *per^01^*;;*U-per*/+ (control, black lines) and *per^01^*;*pdf-G4*/+;*U-per*/+ (pdfPER, blue lines). sLNvs are marked with anti-PDF. TIM and PER images are displayed in NIH ImageJ lookup table 5 Ramps (inverted) for visibility. PDF images used to identify sLNv are in gray scale. Cells (N): (A) sLNv (53–87), (B) LNd (74–122), (C) DN1 (57–208). Average cell intensities were normalized to PPP CT6 = 1 before combining measurements from three (TIM) experiments. In some cases error bars (SEM) are very small and obscured by the data point. In no case were error bars omitted. *p<0.05, **p<0.01, ***p<0.001.

**Figure 5:**
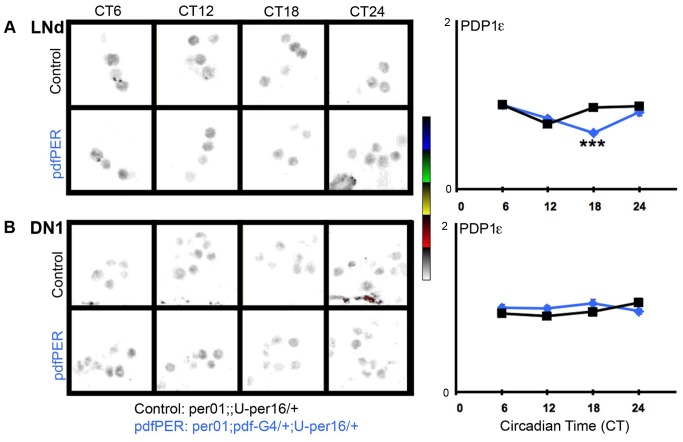
The PDF-cell clock does not impact PDP1ε in non-PDF clock neurons. Labels and nomenclature are the same as in Figure 4. PDP1ε images are displayed in NIH ImageJ lookup table 5 Ramps (inverted) for visibility. Cell group (N): (A) LNd (88–125), (B) DN1 (175–281). *p<0.05, **p<0.01, ***p<0.001.

The ability of PDF neurons to reset molecular clocks in non-PDF neurons has been powerfully demonstrated by selective manipulation of circadian period in PDF neurons and examination of molecular oscillations in non-clock cells [39],[41],[55]. To determine the direct molecular targets of PDF neuron signaling, we rescued clock function selectively in PDF neurons of arrhythmic *per^01^* mutants and assayed molecular oscillations in *per*-less non-PDF neurons. By examining molecular changes in *per^01^* non-PDF neurons, we removed the possibility that identified changes would be indirect through a functioning circadian clock and/or *per*. In addition, we examined molecular changes on the first day of constant darkness, removing the possibility that light signaling via CRY is responsible for any changes. We rescued *per* in PDF cells (*per^01^*;*pdf-G4*/+;*U-per16*/+; “pdfPER” flies) and examined non-PDF-expressing circadian cells (LNd and DN1) in brains stained for the clock components TIM (Figure 4) and PDP1ε (Figure 5). First we demonstrated that transgenically supplied PER cycles in the sLNvs and rescues oscillations in TIM levels and nuclear localization in those cells (CT24), indicating at least a partially rescued sLNv clock consistent with prior studies (Figure 4A) [55].

We then examined the consequences of the rescued sLNv clock on non-PDF *per^01^* DN1 and LNd neurons. Consistent with the fact that these cells lack *per* or a fully functioning circadian clock, TIM is predominantly expressed in the cytoplasm at all times of day (Figure 4) [13]. However we found stark changes in the levels and phase of TIM oscillation in DN1 neurons despite the lack of PER. In *per^01^* controls, we observed a low amplitude TIM oscillation with an inappropriate day-time peak (Figure 4). In pdfPER rescue flies, TIM cycling amplitude increases with elevated peak levels and its oscillation phase is synchonized with that of TIM in the PDF neurons (Figure 4). TIM levels and phase in LNds are also modified by the PDF-neuron clock, but these effects are much smaller than those in the DN1s (Figure 4), consistent with our prior finding that the DN1 are more strongly reset by PDF neurons than the LNd [41]. To determine if these effects on TIM are specific, we also assayed a second clock component PDP1ε, a core circadian transcription factor directly activated by CLK/CYC [3]. We found that PDP1ε exhibits comparable levels between *per^01^* and pdfPER rescue flies with only a modest change at ZT18 in the LNd (Figure 5). The absence of strong effects on PDP1ε suggests that PDF-mediated inputs to the molecular clock are unique to TIM. This result also argues strongly against large PDF effects on CLK/CYC driven transcription, a major determinant of PDP1ε levels [3]. The strength of the effects on TIM evident in the absence of a functioning clock, *per* and light, suggest that TIM is a direct target of PDF signaling.

### Genetic Inhibition of PKA Reduces TIM Protein Levels in the Absence of PER in Non-PDF Neurons

**Figure 6:**
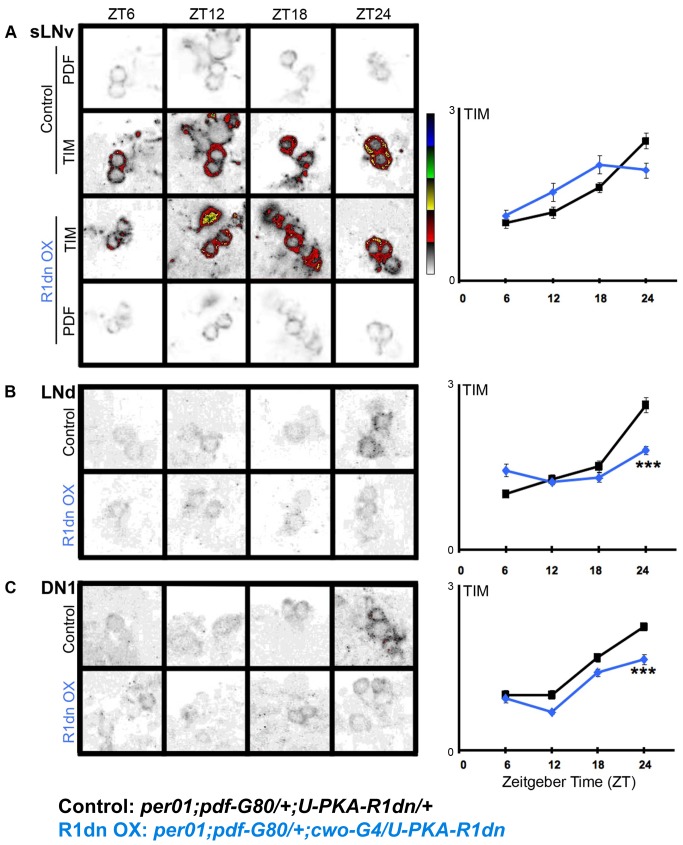
*cwo-G4*>*U-PKA-R1dn* expression reduces TIM levels in the absence of PER in LD. PKA-R1dn was expressed broadly in the circadian system using *cwo-G4* and restricted from PDF cells using *pdf-G80*. Data are displayed as in Figure 4. Cell group (N): (A) sLNv (36–90), (B) LNd (49–130), (C) DN1 (42–195). *p<0.05, **p<0.01, ***p<0.001.

Our results suggest PKA mediates PDF effects and that PDF targets TIM. To test whether PKA can influence TIM levels, we expressed PKA-R1dn in non-PDF circadian cells using *cwo-G4* in combination with *pdf-G80* in a *per^01^* mutant background and examined TIM levels (Figure 6). Flies were entrained and dissected at four time points across the LD cycle, and stained for TIM. We observe reduced peak levels of TIM in both the LNd and DN1 at ZT24 with a non-significant trend developing by ZT12 during the light period. In these *per^01^* flies, TIM remains in the cytoplasm throughout the 24-h cycle. These results indicate that PKA activity positively regulates TIM accumulation in the LNds and DN1s in the absence of a functioning clock suggesting a direct effect on TIM.

### Loss of PDF Reduces TIM Levels in Non-PDF Circadian Neurons

**Figure 7:**
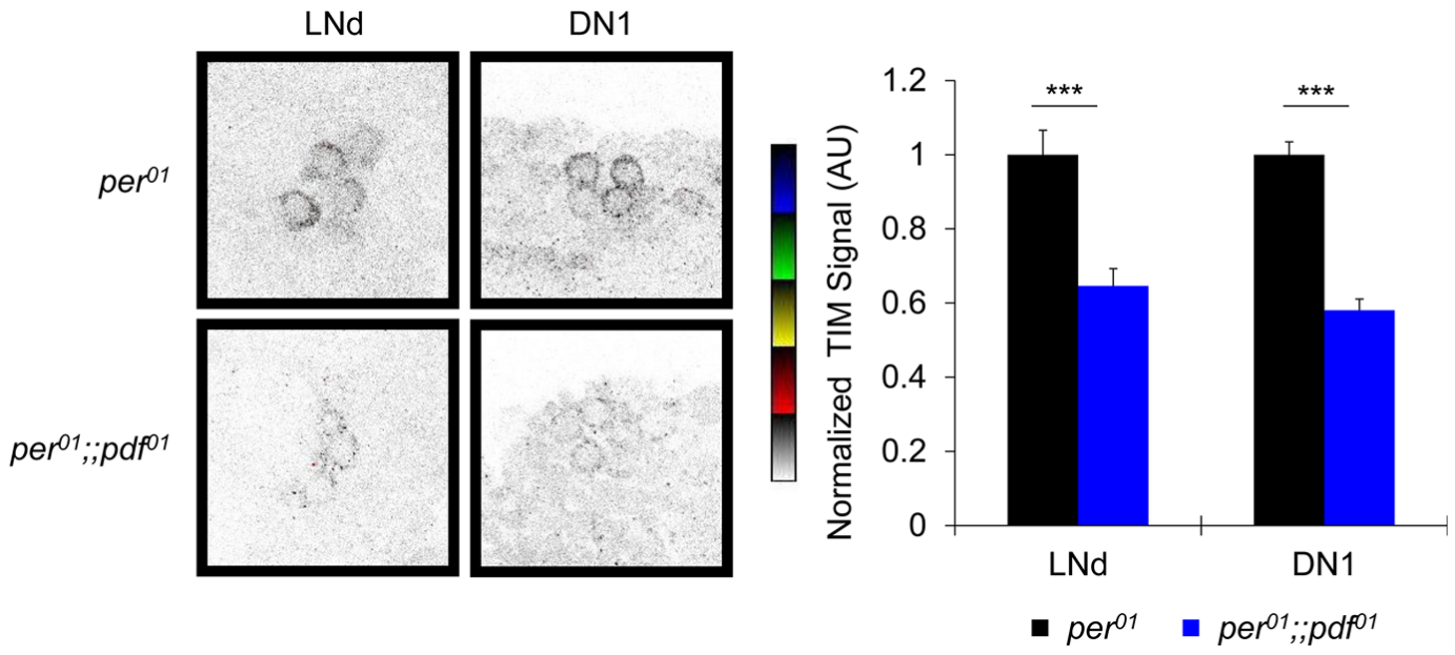
Loss of PDF reduces TIM levels in non-PDF circadian neurons. TIM staining in the *per^01^* mutant background was compared in the presence or absence of endogenous PDF. Brains were collected and fixed at CT24 of DD1. Images are displayed as in Figures 4–6. Bar graphs are normalized TIM staining intensity measurements combined from two independent experiments. Cell group (N): LNd (85–92), DN1 (112–135). ***p<0.001.

We have shown that the PDF-cell clock specifically regulates TIM in non-PDF circadian cells (Figures 4 and 5) and that reducing PKA activity in non-PDF cells leads to reduced TIM levels (Figure 6). Our behavior data show that PKA acts in the PDF signaling pathway downstream of PDFR (Figures 1 and 2). We therefore determined whether loss of PDF would mimic reduced PKA activity and result in reduced TIM. Here we examined loss of PDF (*pdf^01^*) in an arrhythmic *per^01^* mutant background and examined the effects of the PDF peptide on TIM at the end of DD1 (CT24). We compared *per^01^* and *per^01^*;;*pdf^01^* flies and observed that TIM is strongly reduced in the absence of PDF in both LNd and DN1 neurons (Figure 7). We observe a nearly 50% reduction in TIM staining intensity in the absence of PDF, which is comparable to or even larger than the effect than we observed with PKA inhibition. One possibility is that expression of PKA-R1dn may not completely interrupt the signaling cascade, whereas *pdf^01^* is a confirmed null mutation [37]. These results provide further independent support for the hypothesis that the PDF>PDFR>PKA signaling pathway influences the core molecular clock by promoting the accumulation or stability of TIM.

### GFP Reconstitution across Synaptic Partners Defines the DN1p as Direct Targets of PDF+ LNv Neurons

**Figure 8:**
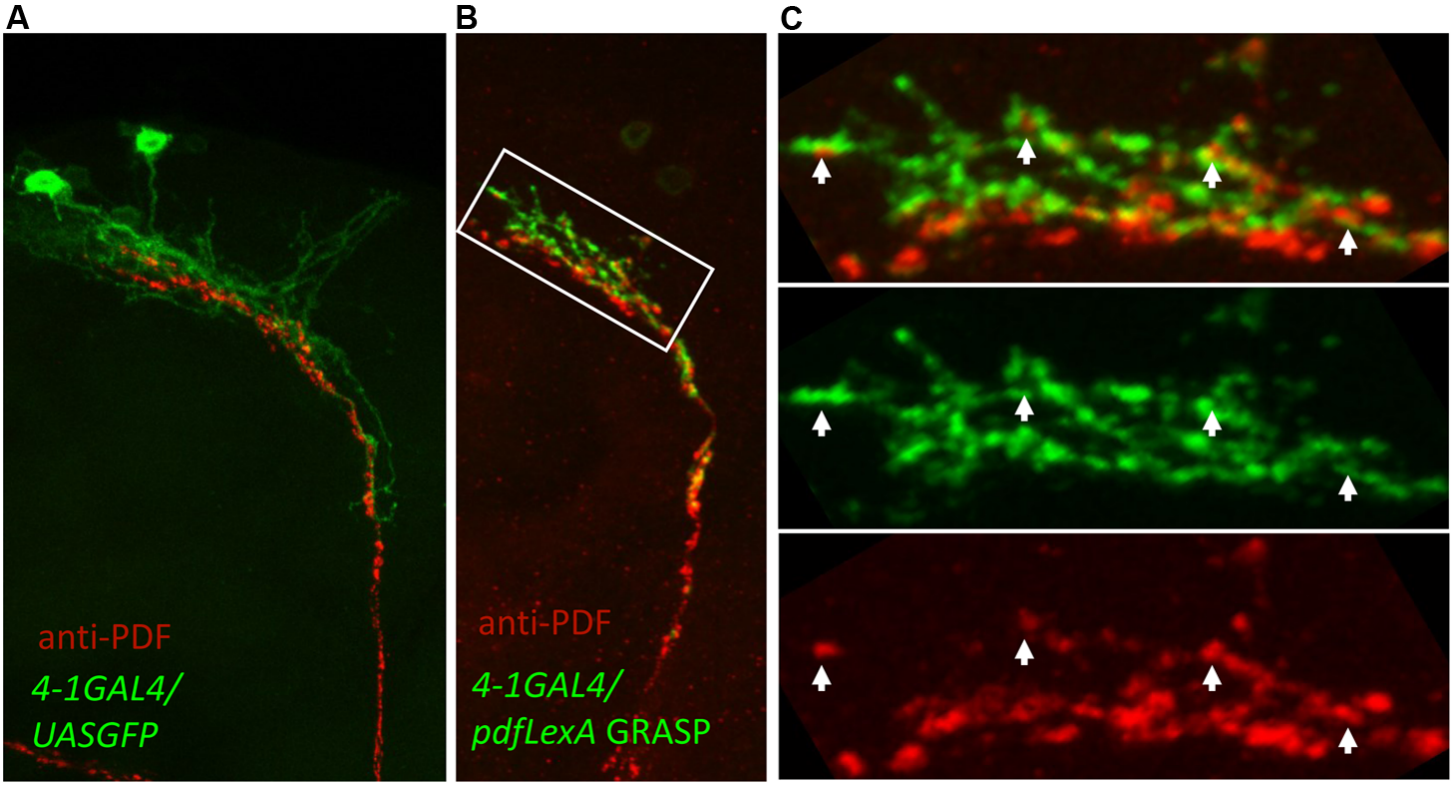
PDF(+) sLNv make direct synaptic connections with DN1p neurons. (A) PDF-labeled sLNv terminals in the dorsal brain (red) are tightly intermingled with DN1p neurites marked by membrane-GFP expression using the *Clk4.1-G4* driver. (B) GRASP labeling (green) demonstrates direct cell-cell contact between the DN1p and anti-PDF labeled sLNv (red). (B) Reconstituted GFP label (green) is observed only at putative points of contact between the two cell groups. (C) Higher magnification of the region outlined in (B) reveals close apposition of the two labels (white arrowheads) but little overlap, delineating the synaptic architecture.

Reduced morning anticipation in *pdfr* mutants coupled to an absence of significant core clock effects in LD suggested that PDF morning function is *via* effects on neuronal output [35]. We hypothesized that these effects may be mediated by direct effects on neuronal activity. Our previous work had identified the DN1p as functional targets of PDF on morning anticipation using rescue of *pdfr* mutants [41]. To determine if this reflects a direct interaction, we selectively labeled the DN1p using *U-GFP* in combination with *Clk4.1-G4* and the LNv using anti-PDF and found that the arbors of each extensively co-mingle (Figure 8A). To test if DN1p neurons are direct targets of PDF neurons, we employed GFP reconstitution across synaptic partners (GRASP) [57]. Here we expressed one fragment of GFP (GFP11) on the extracellular surface of the LNv neurons using *pdf-LexA* and its complementary fragment (GFP1-10) on the extracellular surface of the DN1p neurons using *Clk4.1-G4*. We observe robust fluorescence in the dorsal terminals consistent with extensive physical contacts (Figures 8B, 8C, and S4). Co-labeling of sLNv-DN1p GRASP with PDF finds that PDF signal is in close proximity to GRASP signals suggesting that sites of physical contact are potential release sites for PDF (Figure 8B and 8C).

### PDF Acutely Excites Non-PDF DN1p Neurons

To examine PDF signaling mechanisms that control neuronal output, we first performed live imaging on the DN1p neurons on explanted brains. Using the *Clk4.1-G4* driver in combination with the FRET sensor *U-Epac1*(50A) [58], we measured the variation of [cAMP] following focal PDF application specifically in the DN1p neurons. Prior studies had used bath application of PDF to examine changes in cAMP and thus, the observed effects could be due to indirect activation [47]. Following focal PDF application to the DN1p neurons (Figure S3A), we observed a decrease in the ratio YFP/CFP indicating an increase of [cAMP] (Figure S3B–S3D). Thus, as suggested by prior studies [47], we confirm that PDF increases cAMP levels in the DN1p.

**Figure 9:**
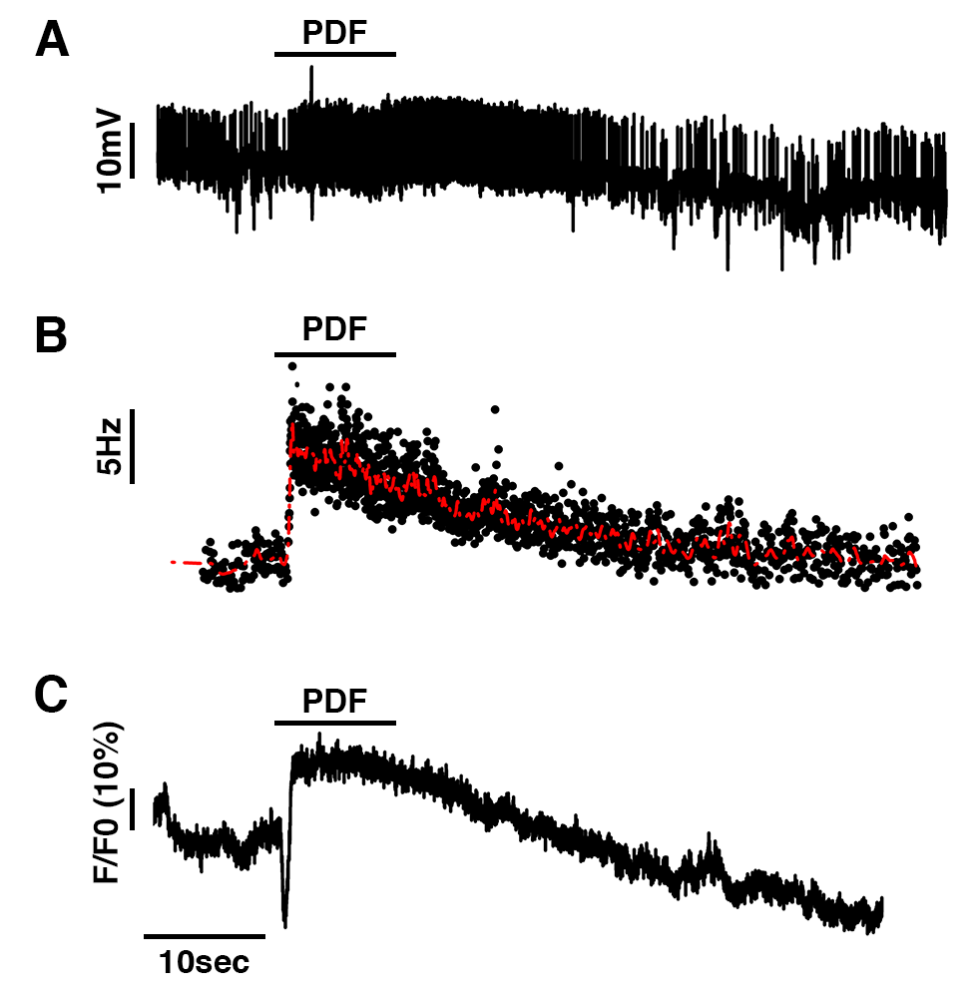
PDF induces an increase in [Ca^2+^]_i_ in the DN1p neurons. Representative current clamp recordings obtained in cell-attached mode showing on the same cell (A) depolarization, (B) increase in instant firing frequency, and (C) increase in [Ca^2+^]_i_ in *U-GcaMP6f*/+;*Clk4.1-G4*/+ male flies (*n* = 3).

To resolve whether PDF acutely controls DN1p neuronal activity, we developed patch clamp electrophysiology of the DN1p subset of pacemaker neurons and assayed the response of these cells to focal PDF application. We performed cell-attached recordings in combination with live calcium imaging on the DN1ps by simultaneously recording firing frequency and [Ca^2+^]_i_ using *Clk4.1-G4* driving expression of the GCaMP6f calcium indicator [59]. Focal PDF application acutely stimulates the DN1ps by increasing the instant firing frequency of the neurons (Figure 9A and B). This increase in neuronal activity is directly correlated with an increase in [Ca^2+^]_i_ as measured by the GCaMP6f calcium indicator (Figure 9C).

**Figure 10:**
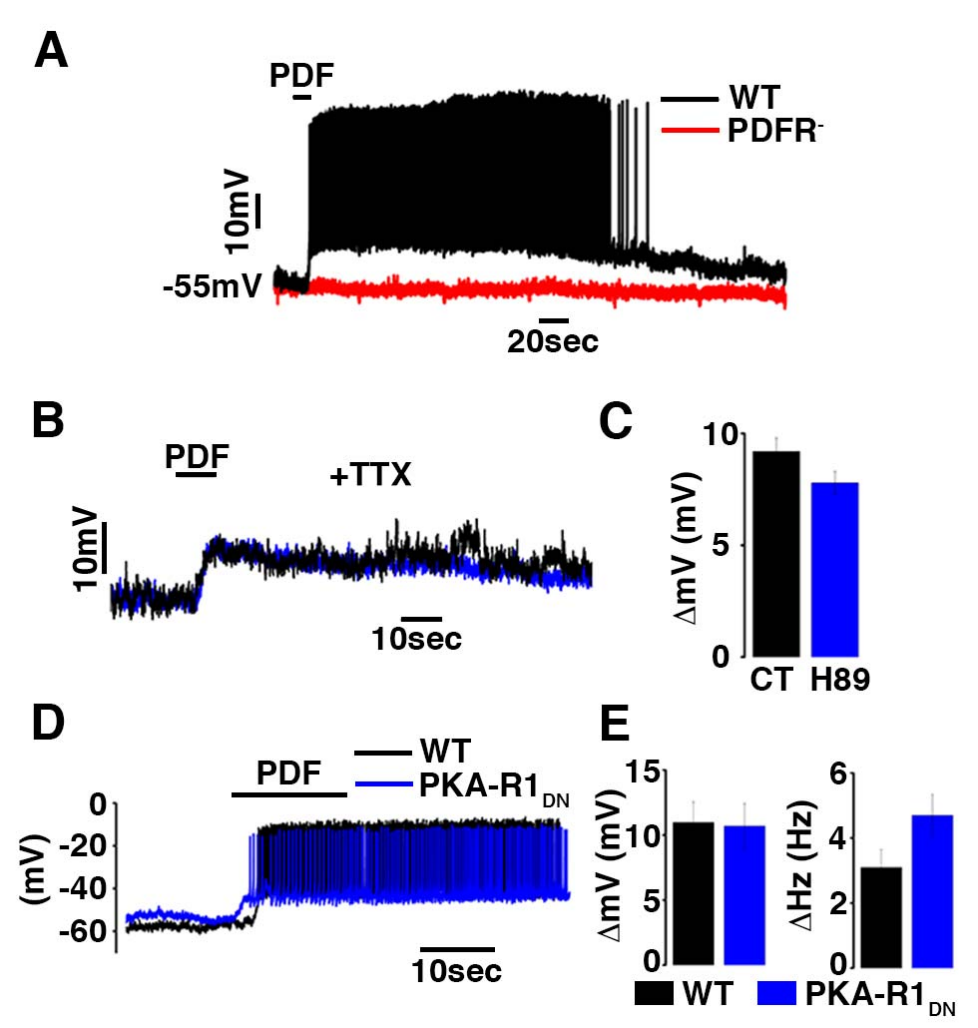
PDF induces depolarization and increase in firing frequency in DN1p neurons. (A) Representative current clamp recordings obtained from UAS-CD8 GFP;Clk4.1M-Gal4 male flies showing the depolarization and increase in firing frequency in control or in PDFR mutant (WT in black, PDFR^−^ in red, *n* = 3). (B) Representative recordings showing the membrane potential in the presence of TTX in control (black, Δ = 9.2±0.6 mV, *n* = 3) or with a PKA inhibitor (H89- blue, Δ = 7.8±0.5 mV, *n* = 3). Mean ± SEM are shown in the histogram (C). (D) Representative recordings showing the PDF induced depolarization control (black, [Δ = 11±1.6 mV, and Δ = 3.4±0.46 Hz *n* = 5]) or in PKA-R1dn (blue, [Δ = 10.7±1.73 mV, and Δ = 4.7±0.64 Hz *n* = 4]). Mean ± SEM are shown in the histogram (E).

Using whole-cell current clamp recordings, we found that PDF both acutely depolarizes and increases action potential firing rates (Figure 10A). This excitatory effect of PDF is dependent on its receptor as we could not detect any effect of PDF on the membrane potential or firing frequency in cells lacking PDFR (Figure 10A). The PDF-evoked depolarization was present after blocking action potential firing, and thus most synaptic transmission, with the voltage gated sodium channel blocker TTX indicating that PDF acts on the DN1ps directly (Figure 10B). Surprisingly the PKA inhibitor H89 did not block these effects indicating that PDF activates a PKA-independent pathway to acutely activate neurons (Figure 10C). To independently confirm the dispensability of PKA signaling we recorded from DN1p neurons expressing the dominant-negative PKA-R1 (Figure 10D and 10E). PDF application still depolarizes and increases in firing frequency further supporting the hypothesis that PDF acts independently of PKA activation to regulate membrane excitability.

**Figure 11:**
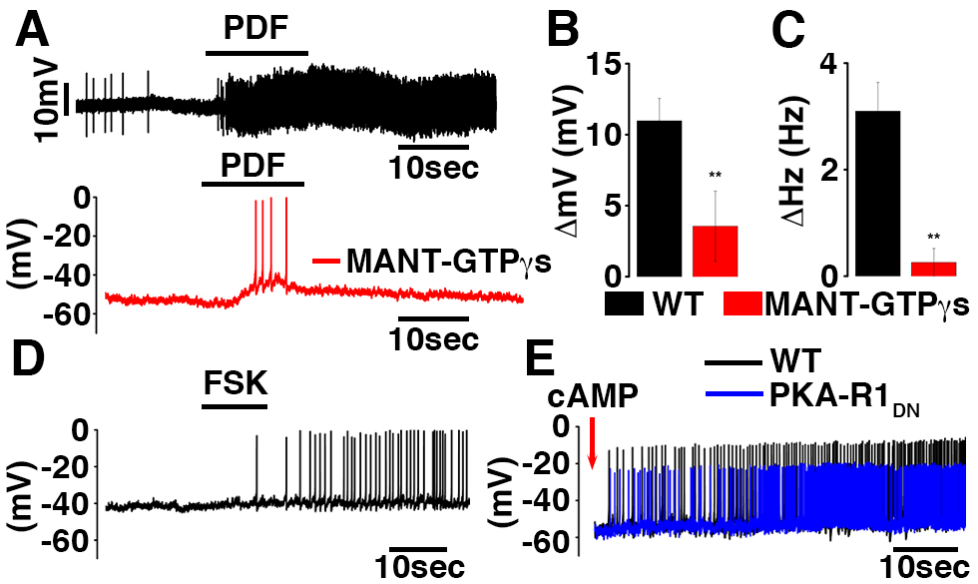
PDF-induced depolarization and increase in firing frequency are dependent on adenylate cyclase. (A) Representative current clamp recordings obtained from the same cell in cell attached configuration first (black trace, before AC inhibitor) and then in whole cell configuration (red trace, after 10 minute dialysis of MANT-GTPγS into the cell). Mean ± SEM are shown in the histogram for changes in the membrane potential (B) and firing frequency (C) (respectively Δ = 11±1.6 mV, and Δ = 3.4±0.46 Hz *n* = 5 in control and Δ = 3.56±2.46 mV, and Δ = 0.26±0.26 Hz *n* = 5 after AC inhibition). Representative current clamp recordings showing the effects of AC activation by application of forskolin 20 µM for 10 s (Δ = 4±0.91 mV, and Δ = 2.28±0.61 Hz *n* = 5) (D) or the effects of cAMP dialysis into the cell in control (black trace, *n* = 3, Δ = 9.6±2.02 mV, and Δ = 5±1.19 Hz) or in PKA-R1dn neurons (blue trace, *n* = 2, Δ = 8.4±2.21 mV, and Δ = 4.6±1.22 Hz) (E). Changes in membrane potential and firing frequency were measured by comparing the first and last 10 s from the 1 minute recording.

We next examined the potential role of cAMP as the intracellular component mediating the acute PDF effect on membrane activity. First, we demonstrated that the adenylate cyclase inhibitor MANT-GTPγS blocks the PDF induced excitation (Figure 11A–11C). Conversely, forskolin (an adenylate cyclase activator) and direct dialysis of cAMP into the cell induces a depolarization similar to the PDF evoked response (Figure 11D and 11E). Finally, the cAMP induced depolarization and activation was present in the neurons expressing PKA-R1dn (Figure 11E). Taken together these data indicate that cAMP, rather than other upstream signaling components is responsible for the PDF effects on excitability.

**Figure 12:**
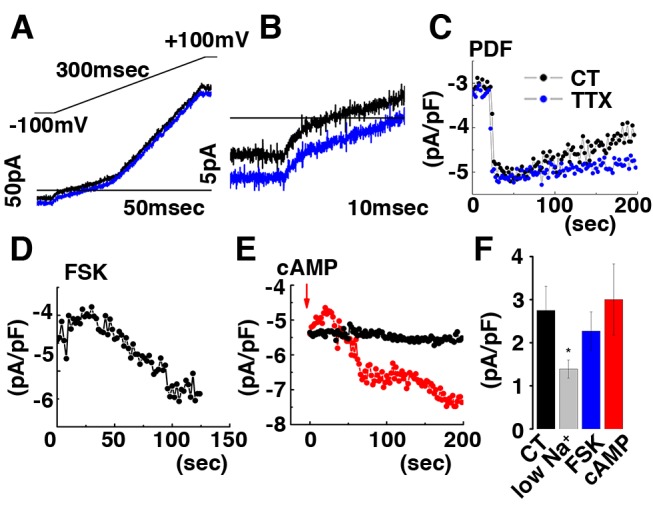
PDF activates a TTX resistant cationic inward current in Drosophila DN1p neurons. (A) Representative voltage clamp recording at ZT6. Changes in ionic currents were measured from a ramp protocol form −100 mV to +100 mV in 300 ms. Black and blue traces represent currents recorded respectively before and after focal application of PDF 50 µM. (B) Crop from (A) showing inward currents from −100 mV to −60 mV. (C) Time course of PDF induced inward rectifying current measured at −100 mV without (black trace) or with TTX 10 µM (blue trace). Time course of FSK (D) or cAMP (E) induced inward rectifying current measured at −100 mV (for (E) black trace is without cAMP dialysis in the pipette, and red trace is when cAMP was added into the intracellular solution). (F) Histograms showing reduced inward current when Na^+^ was replaced from the extracellular solution with NMDG and comparable inward currents in control (CT), forskolin (FSK), or cAMP induced inward currents (respectively, 2.75±0.56 pA. pF^−1^, *n* = 5 in control, 1.39±0.21 pA.pF^−1^, *n* = 3 in low sodium, 2.27±0.44 pA.pF^−1^, *n* = 3 with FSK and 3±0.83 pA.pF^−1^, *n* = 2 in control).

In voltage clamp mode, PDF application acutely induces an inward current at negative potentials and a positive shift in the reversal potential (Figure 12A–12C). This inward current is TTX-insensitive (Figure 12C) and is attenuated after reduction of extracellular sodium (Figure 12F). Furthermore, focal application of the adenylate cyclase activator forskolin or direct intracellular dialysis of cAMP induce an inward current like PDF (Figure 12D and 12E). The properties of the observed current—neuropeptide induction, TTX resistance as well as PKA independence—are consistent with a cyclic-nucleotide-gated channel (CNG) [60]. Thus, using this novel patch clamp analysis and focal application of PDF, we demonstrate that PDF acts as an excitatory neurotransmitter that acutely increases firing rate and calcium, likely in a PKA-independent manner. The rapidity of PDF effects on excitablility argues strongly that they are direct and not via clock resetting.

## Discussion

**Figure 13:**
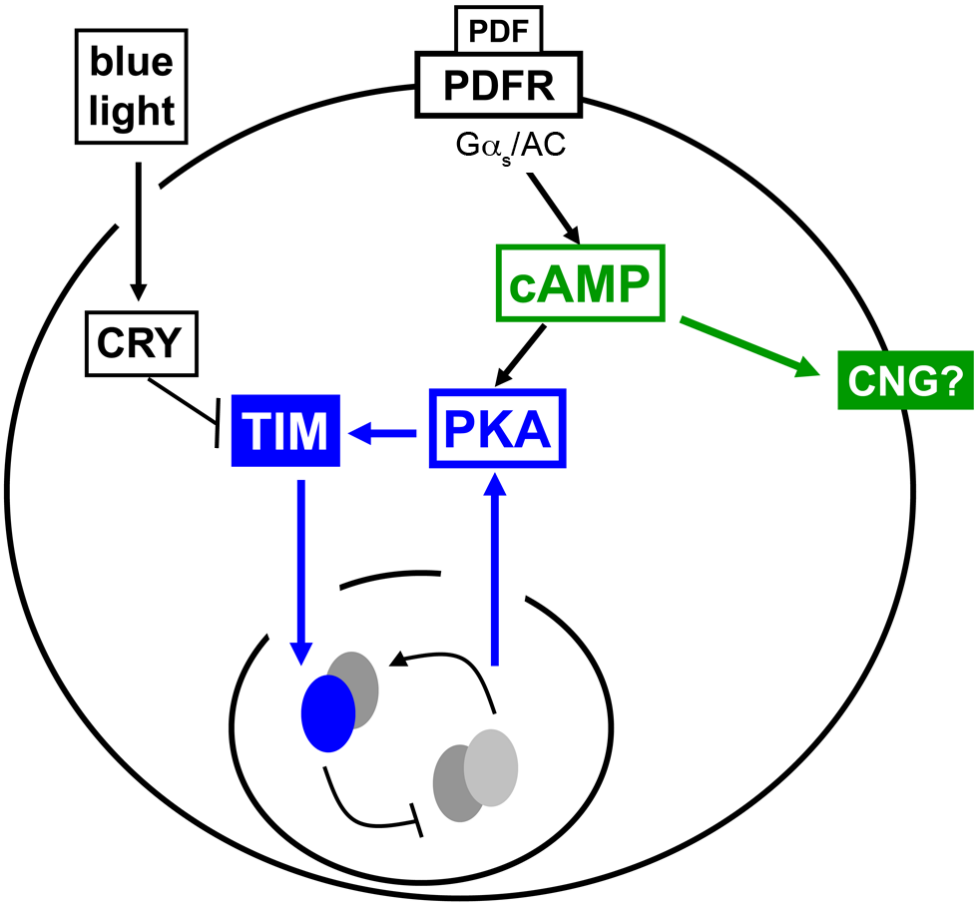
Model for a bifurcation in the PDFR signaling pathway controlling the molecular clock and neuronal excitability. PDFR acts through Gs and adenylate cyclase (AC) to increase levels of cAMP which may directly activate a cyclic-nucleotide-gated (CNG) channel (green pathway) to acutely depolarize the cell and increase the action potential firing rate. cAMP also activates PKA, promoting TIM stability and progression of the molecular clock (blue pathway). Light activates CRY, which promotes TIM degradation. The molecular clock also controls PKA transcripts, thereby controlling signal transduction to the clock through a feedback mechanism.

Intercellular communication has emerged as a critical element in circadian pacemaker function in multicellular animals. PDF acts as a master neural network regulator coordinating molecular oscillations between disparate circadian pacemaker neurons in *Drosophila*. Yet how PDF signaling resets circadian clocks as well as acutely regulates neural activity has not been clearly defined. Here we provide evidence that the PDF signaling pathway works through two mechanisms to regulate circadian behavior; a clock resetting pathway that targets the core clock protein TIMELESS (TIM) via PKA to maintain synchronous molecular oscillations throughout the pacemaker network, and a neural activity pathway that acutely increases the firing rate of pacemaker neurons independent of PKA (Figure 13).

Here we provide *in vivo* genetic evidence for a role for PKA in mediating PDF neuropeptide effects on behavior, including demonstration of clock control of PKA subunits in PDF target neurons. While prior work had demonstrated a role for PKA in circadian behavior, these studies observed effects under conditions of PKA overexpression in mutant conditions [61], failed to link PKA to PDF receptor signaling [49],[61]–[64], or impaired cAMP or PKA throughout the fly [49],[63] or the circadian network [62],[65]. We show that inhibition of PKA in a subset of non-PDF neurons can mimic the advanced circadian activity in the evening observed in PDF or PDF receptor mutants. Moreover, expression of an activated form of PKA can rescue most *pdfr* mutant phenotypes, providing powerful genetic evidence that PKA is mediating PDFR signaling in non-PDF circadian pacemaker neurons (Figure 13).

Using refined cell-specific manipulations, we dissected the functional neuroanatomy of PKA function. These studies demonstrated DN1p PKA contributes to morning anticipation and DD rhythms and PDF(−) sLNv and CRY+ LNd functions in evening anticipation, similar to the division of labor we previously observed for *pdfr* rescue [35],[41]. Nonetheless, the finding of modest effects of DN1p PKA on morning anticipation in the face of a prominent role for non-PDF cells (Table 1) suggest that other non-PDF cells make a contribution and/or that the DN1p function is via PDF effects on neuronal excitability upstream of PKA. While a previous study linked a PDF-coupled adenylate cyclase (AC3) function in the PDF neurons to morning anticipation in sLNv, it is not known if this adenylate cyclase may also couple to other receptors that may mediate these effects, for example, by regulating PDF release rather than PDF receptor signaling [31]. We did observe that PDF neurons also contribute to DD rhythmicity effects of PKA (Table 2), consistent with the more distributed function of PDFR in DD rhythmicity. Taken together, these data provide a circuit map for PKA function in mediating PDF effects in the central pacemaker network.

A central feature of core circadian clock components is their time-of-day dependent expression, providing the mechanistic basis of biological timekeeping. To address whether the clock actively controls PKA expression or activity, we assessed transcript levels using FACS isolation of the DN1p. Here we show that both regulatory subunits (R1 and R2) and one of the three catalytic subunits (C1) of PKA show robust rhythms with a peak during the mid-day. Coordinate oscillations of both regulatory and catalytic subunits of PKA should result in daily increases in the sensitivity to PDFR activation. Rhythmic PKA expression also provides a mechanistic basis for rhythmic behavior under conditions when PDF oscillations are not apparent [29],[56]. PKA rhythms are abolished in mutants of the classical core clock component *per^01^*, indicating these oscillations are clock controlled. Given that these PKA transcripts peak in the mid day (ZT4; Figure 3E–3G) at a time when CLK activity is low [66] and that peak PKA transcript levels are reduced in the *per^01^* mutant suggest that it is not directly CLK-activated. Interestingly, expression of a bacterial sodium channel in the larval sLNv can induce PKA-C1 transcript expression [62], suggesting that clock-driven changes in neuronal activity may mediate PKA transcript rhythms. While the peak of PKA subunit transcription in the DN1p in the mid day is not coincident with the requirement of PDFR signaling for morning anticipatory behavior, the rate of accumulation and half-life of PKA protein in these cells is not known. Regardless, these results indicate that pacemaker neurons rhythmically control their sensitivity to PDF inputs, suggesting that rhythmic PDF-driven cAMP production and rhythmic PKA transcription collaborate to provide a robust time-of-day specific signal to synchronize non-PDF to PDF oscillators (Figure 13).

In addition to demonstrating a key role for PKA in PDFR signaling, we also reveal important *in vivo* evidence that PDF neuronal signaling selectively targets the circadian clock component TIM in non-PDF neurons, providing a molecular basis for network influence on core molecular clocks (Figure 13). To address the core clock target of PDF signaling, we set up a complex genetic scenario in which we used the arrhythmic *per^01^* mutant as a background and rescued *per* (and thus, clock function) only in PDF neurons. We then asked how rescued clock function in PDF neurons impacts molecular clock components in *per^01^* non-PDF clock neurons. We found that these rhythmic PDF neurons are able to drive high amplitude and appropriately phased DN1 molecular oscillations in the core clock component TIM but not in another clock component PDP1ε, suggesting that TIM is the specific target of PDF signaling in the molecular clockwork. We observe reduced effects on TIM in the LNd, perhaps due to the fact that the rescue of *per^01^* in PDF neurons is not complete (Figure 4) [55]. Moreover, it is unlikely that PDF is inducing an intact clock in *per^01^* non-PDF neurons. In addition to the weak or absent PDP1ε oscillations, clock-driven oscillations in TIM nuclear localization are not observed with TIM remaining cytoplasmic, consistent with studies indicating that PER is required for TIM nuclear localization [10],[13]. Loss of PDF in a *per^01^* background results in reduced TIM levels in both the LNd and DN1 (Figure 7). These robust TIM effects in the LNd may reflect a more extreme perturbation of PDF signaling in the null mutant and/or the potential of non-PDF transmitters to influence LNd TIM levels in the PDF cell rescue. Nonetheless, our data support the view that PDF signaling is specifically regulating TIM rather than reconstituting a clock in *per^01^* target neurons.

Our work suggests that PKA is an important intermediary between PDF and TIM (Figure 13). Inhibition of PKA in non-PDF neurons in *per^01^* mutants reduces TIM levels in LNds and DN1s (Figure 6), consistent with a role for PKA in promoting TIM accumulation or stability. The more strongly evident effects of PKA on TIM than in the PDF cell rescue context may reflect the incomplete PDF cell rescue, more robust PKA manipulation with dominant negative expression, and/or PDF or PKA-independent effects of PDF cells on LNd TIM levels. PKA effects on TIM in the absence of *per* or a fully functioning clock suggest that these effects are direct. PKA has also been implicated in activating CLK driven transcription. However, these effects are modest and observed under conditions of PKA overexpression in cultured S2 cells [67]. Moreover, PKA does not phosphorylate CLK *in vitro* [67]. Our finding that PDP1ε in non-PDF cells is not strongly affected by rescue of the molecular clock in PDF neurons further supports the hypothesis that CLK activity is not an *in vivo* target of PDF/PKA. TIM contains numerous consensus PKA phosphorylation sites and is robustly phosphorylated by PKA in vitro [68]. Our findings that reduction of PKA function reduces TIM levels suggests a positive role for PKA in TIM accumulation or stability. Our finding that TIM levels in LNd and DN1 neurons are reduced in the absence of PDF peptide provides strong independent verification for the PDF>PDFR>PKA pathway in targeting TIM to influence the core molecular clock. Notably, we did not observe any effects on TIM in DD as assayed by Western blot after PKA inhibition in the eye (unpublished data), suggesting PKA pathway function in the core clock may be restricted to the pacemaker neurons. Comparable changes in TIM due to light pulses are associated with significant phase shifts [69] that are comparable to, or even exceed, those evening phase effects observed in *pdf* mutants or with PKA-R1dn expression, suggesting that these TIM effects are biologically meaningful.

The finding that TIM responds to PDF and PKA could explain observed interactions between PDF and CRY signaling. Altering the pace of PDF-cell clocks can reset non-PDF clocks. However under standard LD conditions, PDF-cell clocks are only able to reset evening phase after mutation of the CRY photoreceptor, indicating that CRY antagonizes PDFR signaling [70]. Our identification of TIM as a common target of CRY and PDFR signaling provides a plausible mechanism for these phenotypes: CRY-mediated degradation of TIM may render pacemaker neurons insensitive to PDF receptor inputs thus explaining the CRY-dependence of PDF effects on evening phase.

Thus, TIM is a multimodal integrator of core clock, environmental, and network pathways of entraining and maintaining clocks in the pacemaker network: (1) *tim* is transcribed by the CLK/CYC heterodimer and is thus regulated directly by the core feedback loop; (2) TIM protein levels are controlled by environmental light via CRY-mediated degradation; and (3) we demonstrate that TIM responds to network signals via PDF signaling, likely directly mediated post-transcriptionally by PKA.

In addition to elucidating signaling mechanisms that link PDF to core clocks, we also defined mechanisms by which PDF acutely regulates neuronal activity. While our work suggests that PDF acts via changes in protein abundance to reset clocks, our previous work suggested that PDF also has effects on pacemaker neuron output, specifically morning behavior, that are independent of resetting clocks [35]. In fact, PDF injection in cockroaches acutely regulates neuronal activity [45]. However, the precise nature and mechanism by which PDF achieves these effects are not clear. Here we have developed patch clamp electrophysiology of the DN1p subset of neurons and assayed the response of these cells to focal PDF application. We focally applied PDF to these neurons and found that PDF both acutely depolarizes and increases action potential firing rates in a PDFR dependent manner, indicating that PDF is acutely excitatory and providing a mechanistic basis for effects on pacemaker neuron output (Figure 9A). Consistent with our data in the DN1p, membrane-tethered PDF peptide expressed in the PDF+ LNv depolarizes the sLNv [29]. Surprisingly PKA inhibiton (by H89 or the expression of a dominant negative PKA) did not block these effects, while adenylate cyclase inhibition did block them, indicating that PDF activates a cAMP-dependent, PKA-independent pathway to acutely activate neurons (Figures 10B–10E and 11A–11E). We note that genetic inhibition of PKA in the DN1ps only modestly reduces morning anticipation, suggesting a potential role for this PKA-independent pathway in morning behavior (Figure 3B and 3C). Given the properties of the PDF-induced current we hypothesize that PDF-driven cAMP activates a cyclic nucleotide gated channel to acutely depolarize and activate target neurons (Figure 13). Our model is consistent with the role of G-alpha-s and cAMP in mediating PDF effects in the sLNv on morning and evening activity allocation [29]. However, the role of PKA was not examined this study.

While we cannot exclude the possibility of direct or indirect cross talk between pathways, these data reveal a bifurcation of the PDF receptor signaling pathway: a PKA-dependent fork contributes to synchronization of the molecular clocks via regulation of TIM and a PKA-independent fork acutely induces neuronal activity (Figure 13), thus providing mechanistic bases for the dual functions of PDF in the *Drosophila* circadian pacemaker network.

## Materials and Methods

### Fly Strains

Fly lines carrying U-PKA-mC* and *U-PKA-R1dn* (also known as BDK33) were a generous gift from Daniel Kalderon [52]. *UAS-CD4::spGFP1-10* and *LexAop*-*CD4:spGFP11* flies were the gift of Kristen Scott [71]. The latter transgene was recombined with *pdf-LexA* (a gift of Michael Rosbash [72]). Lines carrying combinations of these and other transgenes or mutants were constructed using standard genetic crosses. *Tim* was genotyped for the *s/ls* alternative start site polymorphism using previously described primers (Peschel 2004). TIM staining in the *per^01^*;*pdf-G4*;*U-per16* rescue context was completed three times: in two trials the *tim* genotypes were *s/ls*, and in one trial the control was *ls/ls*, while the pdfPER flies were *s/ls*. The staining results were comparable between these conditions and were combined. Strains for TIM staining with PKA-R1dn expression (Figure 6) were *s/ls*.

### Behavior Analysis

Fly behavior was recorded using the Drosophila Activity Monitoring system (Trikinetics) and analyzed using ClockLab and the Counting Macro as described [73]. Briefly, male flies were fed on 5% sucrose-agar medium in 5LD7DD conditions at 25C. LD eductions were obtained using averaged data in 30-minute bins across days 2–5 of the behavior run. DD period and rhythmicity data were calculated in ClockLab with period measurements taken only from flies in which the Power-Significance (P-S) ≥10.

Morning anticipation was calculated using a variant of the method described in [32]. Activity from each of four days of LD behavior recorded for each individual fly were analyzed such that the morning index (MI) = ((total activity 3 h prior to lights-on)/(total activity 6 h prior to lights-on)) − (0.5). 0.5 was subtracted so flat activity over the six hours analyzed is equal to 0. In cases where no activity counts occurred in the 6 hours before lights-on, resulting in an undefined 0/0, the ratio was set to 0.5, indicating no change in activity over that time period.

The timing of evening activity onset was calculated as previously described [35] with the onset time defined as the first time point in the four 30-min bin sliding window with the largest increase in activity prior to lights-off.

Genotypes were compared by Student's two-tailed t-test.

### Whole-Mount Brain Staining

Flies to be stained were entrained for five to seven 12-h light, 12-h dark (LD) cycles at 25°C and either dissected and fixed at the indicated timepoint for LD staining (Figure 6) or transferred to constant darkness and dissected and fixed for DD1 staining (Figures 4 and 5). Brains were dissected in PBS (pH 7.5) and fixed in 3.7% formaldehyde in PBS for 1 h shaking at room temperature. Brains were then washed 3× in PBS and primary antibody solution was added. Guinea pig anti-TIM (1∶2,000), rabbit anti-PER (1∶16,000), and mouse anti-PDF (1∶500) (Developmental Studies Hybridoma Bank) were incubated overnight shaking at 4°C in a solution of PBS, 10% goat normal serum (GNS), and 0.3% Triton X-100. For stains involving rabbit anti-PDP (1∶200) brains were dissected and fixed as above, except after fixation and 3× washes with PBS, brains were subject to a 1-h permeablization in PBS +1% Triton X-100 and primary antibody solution was incubated for 3 days in PBS with 0.3% Triton X-100 and 10% GNS. After the primary incubation, for all stains, brains were washed 3× in PBS +0.3% Triton X-100 and secondary antibodies (for PDF, PER, TIM staining: anti-mouse Alexa647, anti-guinea-pig Alexa 488, anti-rabbit Alexa 594; for PDF, PDP1ε staining: anti-mouse Alexa 594, anti-rabbit Alexa 488) (all dyes from Molecular Probes - Invitrogen) were each added at 1∶500.

### Quantitative Image Capture and Analysis

Brain images were taken on a Nikon E800 laser-scanning confocal microscope using a 60× A 1.40 N.A. objective with laser, filter, and gain settings remaining constant within each experiment. Channels were scanned sequentially. Confocal Z-stacks were analyzed in NIH ImageJ software. Intensity measurements were taken from single confocal sections at approximately the middle of each cell. Nearby areas of similar area to the cells being measured were selected for each cell group in each hemisphere as a measurement of background staining. The background measurement for each cell group in each hemisphere was subtracted from the intensity measurement for each cell in that group. Background-subtracted values were then averaged across all brains in that experiment. Image measurements were normalized prior to combining data from independent experiments. For Figures 4 and 5, each experiment was individually normalized such that pdfPER Rescue at CT6 = 1. For Figure 6, data were normalized to Control at ZT6 = 1. For Figure 7, data were normalized to *per^01^* at CT24 = 1. Data from independent experiments were combined post-normalization to obtain the final graphs. Images from Figures 4, 5, and 6 are displayed using the inverted 5 Ramps lookup table within ImageJ for ease of viewing images with low signal. Staining data were statistically analyzed by one-way ANOVA and Tukey's pairwise comparisons.

GRASP signal and mouse anti-PDF stained brains were fixed, mounted, and imaged as above, except using anti-mouse Alexa 594 to label PDF. Both 40× and 60× objective images were collected in 1 micron steps through the region containing staining, or through the entire dorsal brain for non-labeled parental control lines.

### Fluorescence-Activated Cell Sorting of DN1p Neurons

Cells were processed as described previously [74]. Before FACS cell sorting cells were filtered using 100 micron filter. Propidium iodide (Sigma, 130 ng/ul) was added to distinguish between dead and alive cells. Cells were sorted on Aria II FACS Cell Sorter (BD Biosciences) into an extraction buffer from the PicoPure RNA extraction kit (Arcturus). Transcripts were obtained from 40 to 45 brains (yielding 300–500 DN1p neurons) per time-point. Subsequently, the cells were lysed and stored at −80°C until RNA extraction as described previously [74].

### Transcript Analysis by Quantitative RT-PCR

Cells were processed as described previously [75]. cDNA from two independent replicates per genotype were analyzed per time-point on a BioRad CFX384 real-time PCR system. mRNA was quantified as described previously [74]. One-way ANOVA was used to determine statistically significant differences between time-points within each genotype (*p*<0.05). The following primers were used to examine *pka* expression: *pka-R1*, F primer, 5′-ACTTTGGCGAGATTGCTCTG-3′; R primer, 5′-CGGACAACGATACGAAACTG-3′; *pka-R2*, F primer, 5′-CTACGAACGCATGAATCTGG-3′; R primer, 5′-GCCGAAGTACTGTCCCTTGC-3′; *pka-C1*, F primer, 5′-ATCGCTGGCATCGTAGTCG-3′; R primer, 5′-AAGGCGCTTGGTTAAGACG-3′.

### Electrophysiological Recordings from DN1p Neurons

Brains from male adults Drosophila (7–14 days old) were removed from their heads in ice-cold recording solution. After removing the connective tissue, air sacs, and trachea with fine forceps, the brains were transferred to a recording chamber and were held ventral side down by a harp slice grid (ALA scientific). No enzymatic treatment was used to avoid altering ion channels function on the cell surface. Brains were allowed to rest in continuously flowing oxygenated saline (95% oxygen and 5% carbon dioxide) for at least 10 min and no more than 2 h before recording. Perfusion with oxygenated saline was continued throughout the recording period. Whole brain electrophysiology and imaging experiments were performed on an Ultima two-photon laser scanning microscope (Prairie Technologies) equipped with galvanometers driving a Coherent Chameleon laser. Fluorescence was detected with photomultiplier tube. Images were acquired with an upright Zeiss Axiovert microscope with a 40×0.9 numerical aperture water immersion objective at 512×512 pixel resolution and 1-µm steps. Current-clamp recordings were performed with pipettes (10–14 MΩ) filled with internal solution. To visualize the recorded cell, Alexa Fluor 594 biocytin (10 µM) was added into the intracellular solution. Recordings were made using Axopatch 200B patch-clamp amplifier, Digidata 1320 A, and pCLAMP software (Axon Instruments). The extracellular recording solution contains in mM: 101 NaCl, 1 CaCl_2_, 4 MgCl_2_, 3 KCl, 5 glucose, 1.25 NaH_2_PO_4_, and 20.7 NaHCO_3_ (pH 7.2, 250 mOsm). The internal solution contains in mM: 102 K-gluconate, 0.085 CaCl_2_ 1.7, MgCl_2_, 17 NaCl, 0.94 EGTA, 8.5 HEPES, 4 Mg-ATP, 0.3 Tris-GTP, and 14 phosphocreatine (di-tris salt) (pH 7.2, 235 Osm). For simultaneous cell attached and live calcium-imaging recordings, the Drosophila DN1ps neurons were visualized with GCaMP6f indicator [59]. The x-y images of GCaMP6f fluorescence were acquired at 10–20 Hz. GCaMP6f fluorescence was excited at 840 nm and was captured at wavelengths between 490 and 540 nM using a bandpass filter.

### Live cAMP Imaging from *Drosophila* DN1p Neurons

Changes in intracellular cAMP concentration were imaged using the Epac1-cAMPs indicator. Whole brain imaging experiments were performed using hemolymph-like HL3 saline [76] (in mM: NaCl 70, KCl 5, CaCl_2_ 1.5, MgCl_2_ 20, NaHCO_3_ 10, D-trehalose dihydrate 5, sucrose 115, Hepes 5, pH adjusted at 7.1 with NaOH 1 M). After dissection, whole brains were placed with HL3 solution in the experimental chamber (POC-R perfusion chamber, Zeiss) and placed on the stage of an Axiovert 200 M inverted microscope attached to a Zeiss 510 Meta/ConfoCor3 Laser Scanning unit (Zeiss) available through the Northwestern University Biological Imaging Facility. The x-y confocal images of Epac1-camps fluorescence were acquired at 2–4 Hz using a Zeiss planApochromat 20×0.8 N.A. objective. Epac1-camps fluorescence were excited at 454 nm by a 200 mW argon ion laser and were captured at wavelengths between 470 and 500 nM for CFP and between 510 and 550 nM for YFP using a bandpass filter. The pinhole was set to provide a confocal optical slice of 10 µm. Epac1-camps fluorescence intensity was normalized to the average fluorescence intensity in the images captured before neurotransmitter application and the ratio YFP/CFP was calculated.

### Chemicals

PDF (50 µM, dissolved in recording solution, GenScript) or forskolin (Sigma) was applied focally for 10 s to the recorded cells *via* pressure ejection (0.5–1 psi) from a glass pipette (5–10 µM) placed in the vicinity of the cell. TTX (Tocris) and/or H89 (Sigma) were bath applied by exchanging the recording solution. cAMP (10 µM, Sigma) and MANT-GTPγS (500 nM, Sigma) were diluted into the intracellular solution.

## Supporting Information

Figure S1 **Expression pattern of** ***pdf-G80*** **/+;** ***cwo-G4*** **/+.** (A and B) Maximum projections of confocal sections taken in representative adult *pdf-*G80/*+;cwo*-G4/*U*-*nGFP* (green) brains labeled with anti-PER antibody (red). Sections contain either the LNs (A) or the DNs (B). LN and DN subgroups are indicated by lines.
(PDF)

Figure S2 **PKA-R1dn expression in CRY+ LNd and 5^th^ sLNv phase-advances evening activity onset with no effect on morning behavior.** Graphs, quantification, and overlays are as in Figure 1. Genotype (N). (A) mai179-G4/+;pdf-G80/+ (31), (B) mai179-G4/+;pdf-G80/U-PKA-R1dn (26). *p<0.05 versus both parental controls.
(PDF)

Figure S3 **Epac FRET response to PDF in DN1p neurons.** (A) Representative images showing whole brain imaging using UAS-EPAC;Clk4.1M-Gal4 male flies. Changes in YFP and CFP fluorescence were measured in the region of interest delineated by the blue, red, and green lines. (B) Representative images (5-s interval between images) showing the change in the ratio YFP/CFP after PDF is applied (+0 s). Time course of the ratio YFP/CFP is shown in (C) for the three region of interest delineated in (A). (D) Average ratio YFP/CFP with SEM in black, YFP in yellow, and CFP in blue. (Δratio = 0.13±0.05, *n* = 19 cells from four brains).
(PDF)

Figure S4 **GRASP labeling is absent in parental strains.** (A) When both fragments of GFP are expressed, one using GAL4-UAS and the other with LexA-LexOp, GFP expression is detected at points of contact between cells expressing the two constructs. (B) Parental lines expressing only one fragment of the GFP lack expression in the same region.
(PDF)

## Abbreviations

DD: constant darkness
FACS: fluorescence-activated cell sorting
GRASP: GFP reconstitution across synaptic partners
LD: light-dark
lLNv: large ventral-lateral neuron
PKA: protein kinase A
P-S: power-significance
sLNv: small ventral-lateral neuron
