# Supplementary material for: Dual PDF Signaling Pathways Reset Clocks Via TIMELESS and Acutely Excite Target Neurons to Control Circadian Behavior
Source: PLoS Biol. 2014 Mar 18;12(3):e1001810. doi: 10.1371/journal.pbio.1001810 (PMC3958333; doi:10.1371/journal.pbio.1001810)
Supplement: Figure S1 — Expression pattern of pdf-G80 /+; cwo-G4 /+. (A and B) Maximum projections of confocal sections taken in representative adult pdf-G80/+;cwo-G4/U-nGFP (green) brains labeled with anti-PER antibody (red). Sections contain either the LNs (A) or the DNs (B). LN and DN subgroups are indicated by lines. (PDF) [file pbio.1001810.s001.pdf]

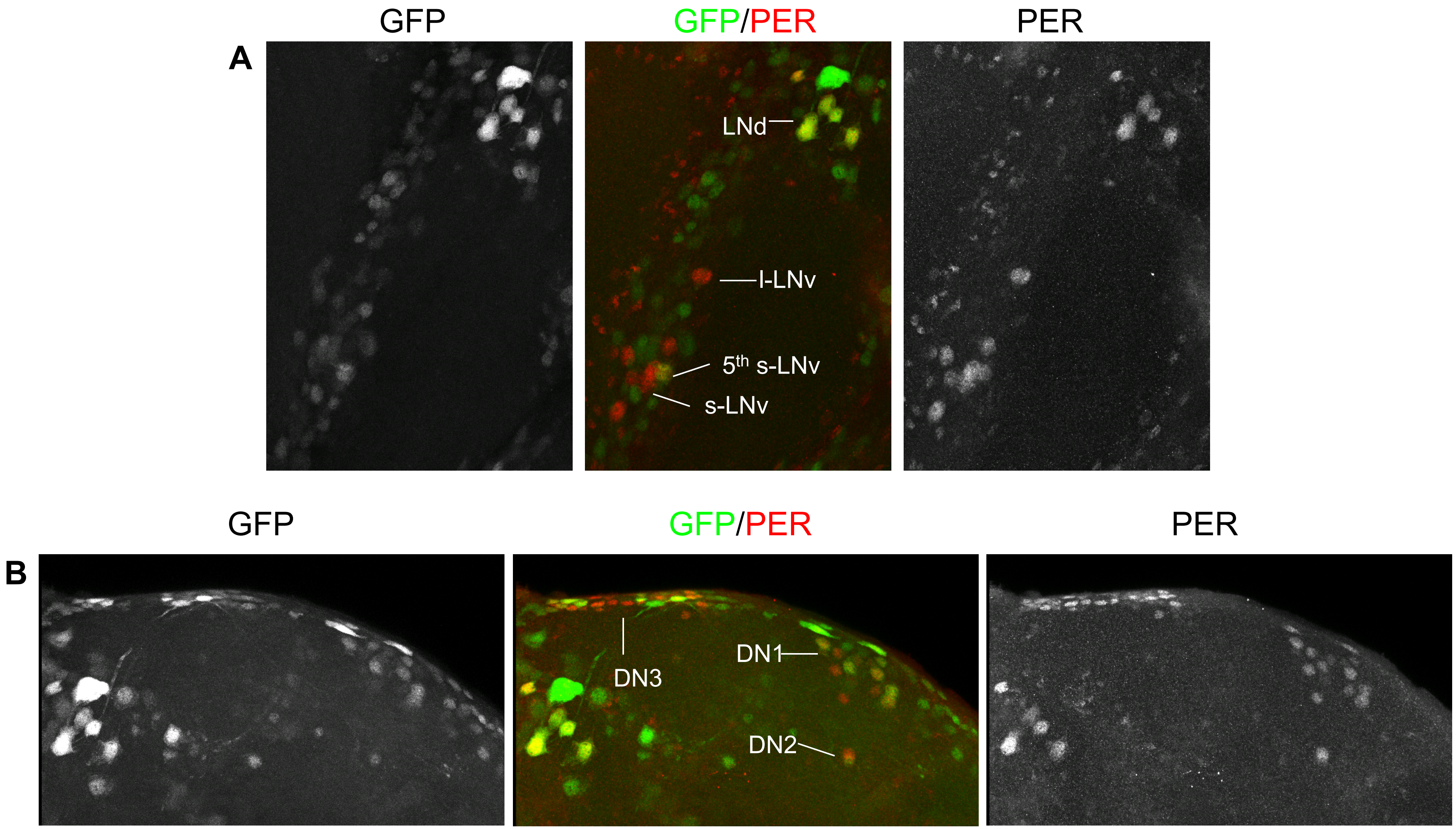

Supplementary Figure 1: Expression pattern of *pdf-G80*/+;*cwo-G4*/+
A and B: Maximum projections of confocal sections taken in representative adult *pdf*-G80/+;*cwo*-G4/*U-nGFP* (green) brains labeled with anti-PER antibody (red). Sections contain either the LNs (A) or the DNs (B). LN and DN subgroups are indicated by lines.
