# Supplementary material for: Dual PDF Signaling Pathways Reset Clocks Via TIMELESS and Acutely Excite Target Neurons to Control Circadian Behavior
Source: PLoS Biol. 2014 Mar 18;12(3):e1001810. doi: 10.1371/journal.pbio.1001810 (PMC3958333; doi:10.1371/journal.pbio.1001810)
Supplement: Figure S2 — PKA-R1dn expression in CRY+ LNd and 5th sLNv phase-advances evening activity onset with no effect on morning behavior. Graphs, quantification, and overlays are as in Figure 1. Genotype (N). (A) mai179-G4/+;pdf-G80/+ (31), (B) mai179-G4/+;pdf-G80/U-PKA-R1dn (26). *p<0.05 versus both parental controls. (PDF) [file pbio.1001810.s002.pdf]

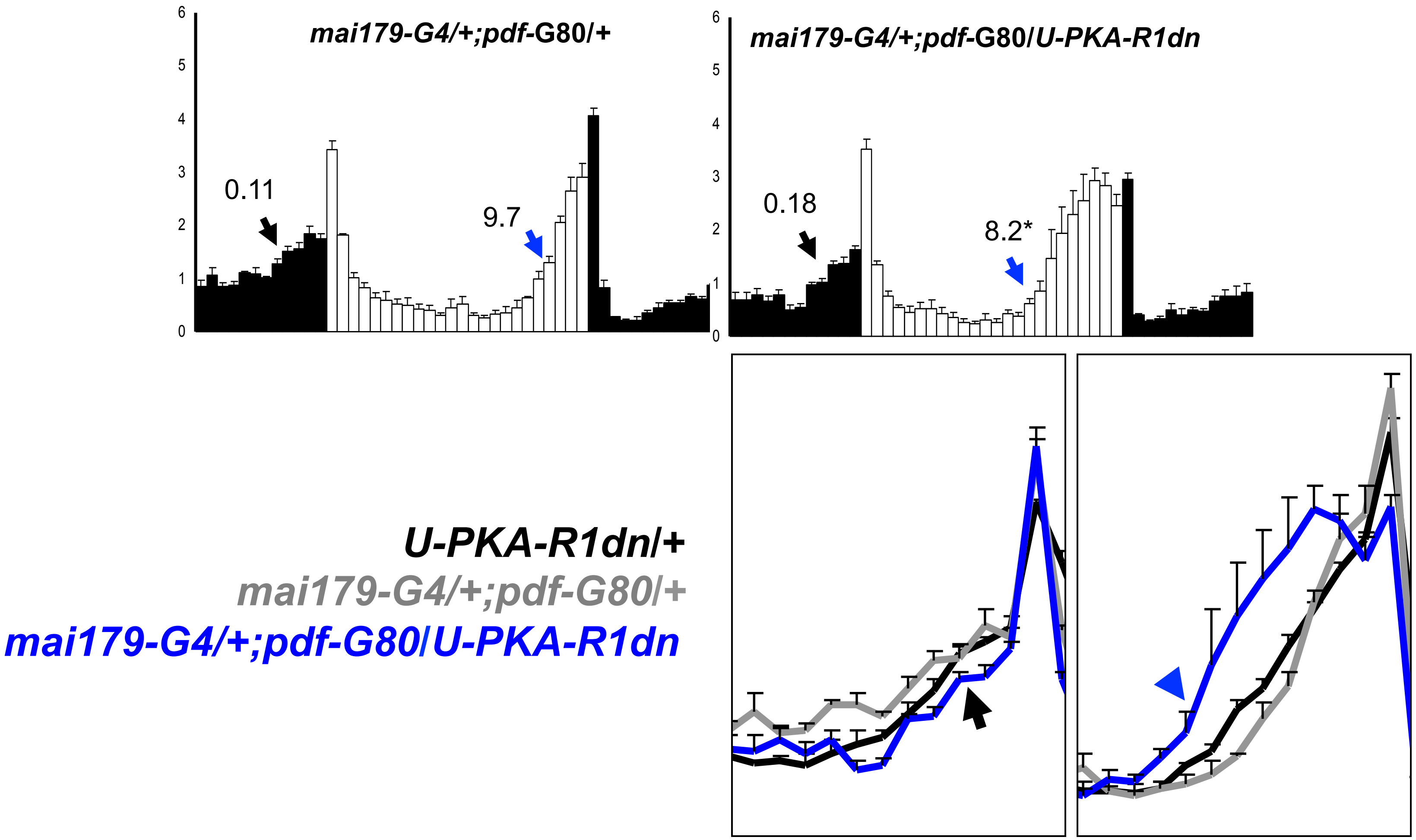

Supplementary Figure 2: PKA-R1dn expression in CRY+ LNd and 5th sLNv phase-advances evening activity onset with no effect on morning behavior.

Graphs, quantification and overlays are as in Figure 1.

Panel: Genotype (N)

A: mai179-G4/+;pdf-G80/+ (31), B: mai179-G4/+;pdf-G80/U-PKA-R1dn (26)

*p<0.05 vs both parental controls.
