# Supplementary material for: Dual PDF Signaling Pathways Reset Clocks Via TIMELESS and Acutely Excite Target Neurons to Control Circadian Behavior
Source: PLoS Biol. 2014 Mar 18;12(3):e1001810. doi: 10.1371/journal.pbio.1001810 (PMC3958333; doi:10.1371/journal.pbio.1001810)
Supplement: Figure S3 — Epac FRET response to PDF in DN1p neurons. (A) Representative images showing whole brain imaging using UAS-EPAC;Clk4.1M-Gal4 male flies. Changes in YFP and CFP fluorescence were measured in the region of interest delineated by the blue, red, and green lines. (B) Representative images (5-s interval between images) showing the change in the ratio YFP/CFP after PDF is applied (+0 s). Time course of the ratio YFP/CFP is shown in (C) for the three region of interest delineated in (A). (D) Average ratio YFP/CFP with SEM in black, YFP in yellow, and CFP in blue. (Δratio = 0.13±0.05, n = 19 cells from four brains). (PDF) [file pbio.1001810.s003.pdf]

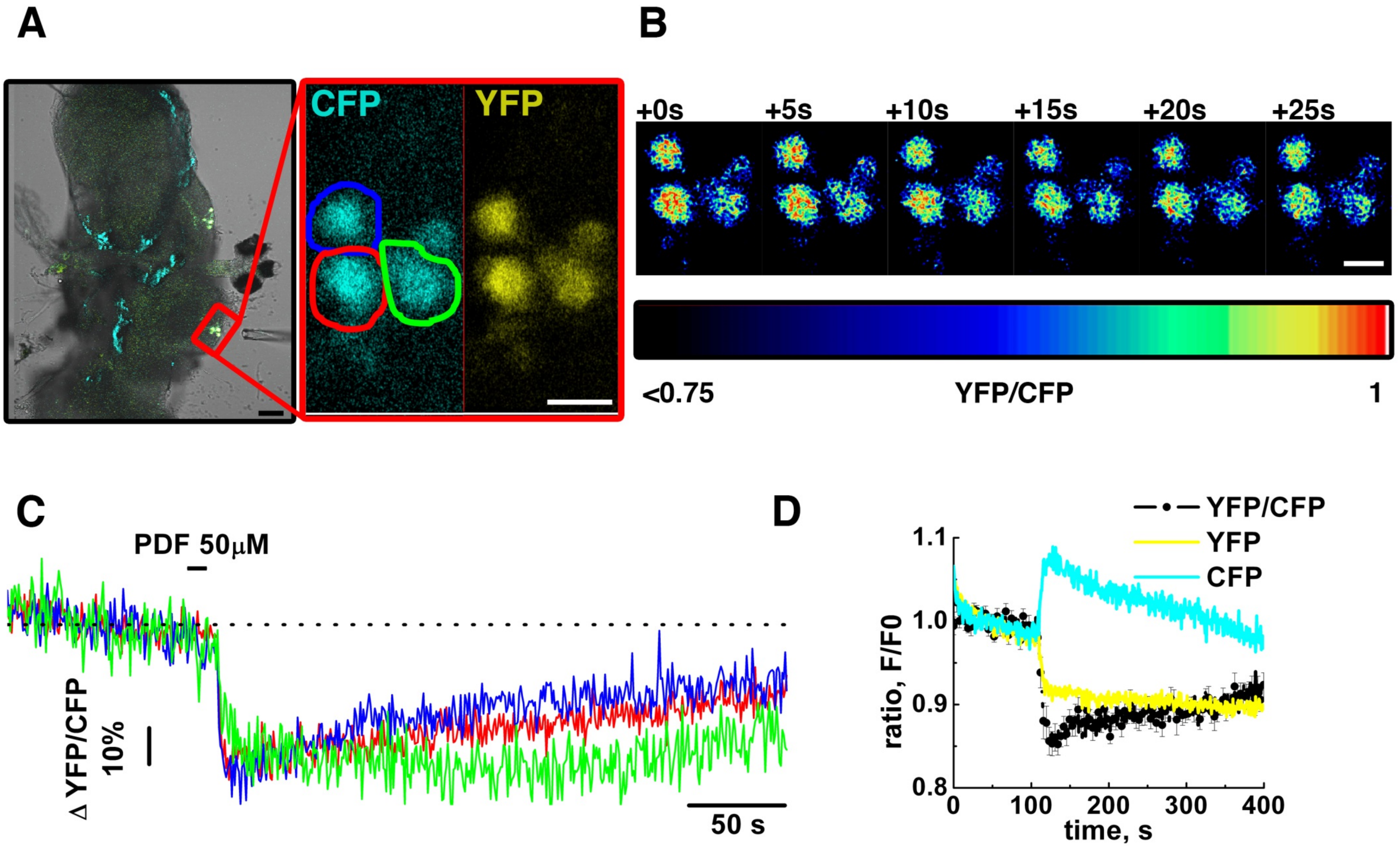

Supplementary Figure 3: Epac FRET response to PDF in DN1p neurons

A: Representative images showing whole brain imaging using UAS-EPAC;Clk4.1M-Gal4 male flies. Changes in YFP and CFP fluorescence were measured in the region of interest delineated by the blue, red and green lines. B: Representative images (5s interval between images) showing the change in the ratio YFP/CFP after PDF is applied (+0s). Time course of the ratio YFP/CFP is shown in C: for the three region of interest delineated in A. D: Average ratio YFP/CFP with SEM in black, YFP in yellow and CFP in blue) (Δratio=0.13±0.05, n=19 cells from 4 brains).
