# Supplementary material for: Dual PDF Signaling Pathways Reset Clocks Via TIMELESS and Acutely Excite Target Neurons to Control Circadian Behavior
Source: PLoS Biol. 2014 Mar 18;12(3):e1001810. doi: 10.1371/journal.pbio.1001810 (PMC3958333; doi:10.1371/journal.pbio.1001810)
Supplement: Figure S4 — GRASP labeling is absent in parental strains. (A) When both fragments of GFP are expressed, one using GAL4-UAS and the other with LexA-LexOp, GFP expression is detected at points of contact between cells expressing the two constructs. (B) Parental lines expressing only one fragment of the GFP lack expression in the same region. (PDF) [file pbio.1001810.s004.pdf]

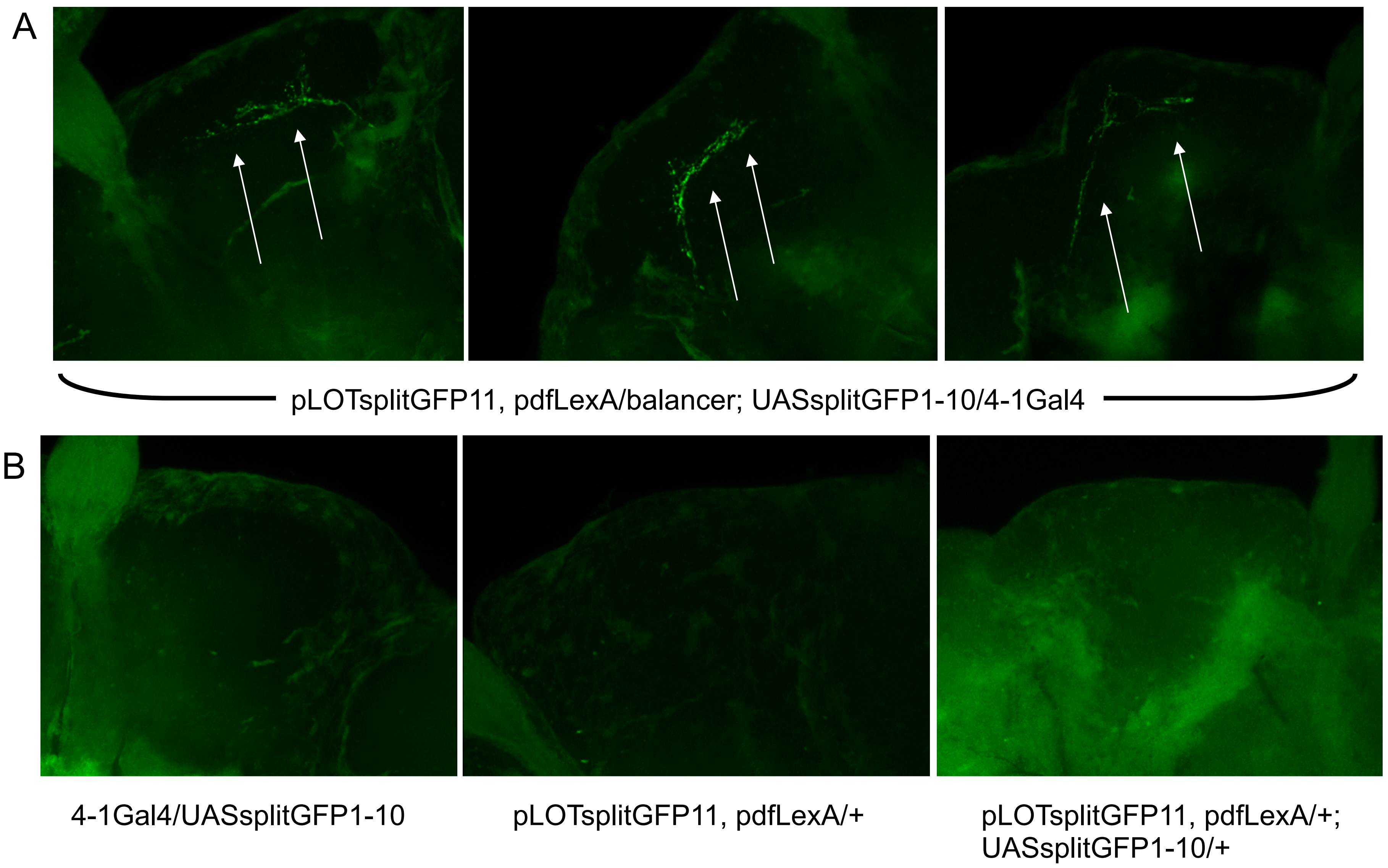

Supplemental Figure 4. GRASP labeling is absent in parental strains.
A:When both fragments of GFP are expressed, one using GAL4-UAS and the other with LexA-LexOp, GFP expression is detected at points of contact between cells expressing the two constructs. B: Parental lines expressing only one fragment of the GFP lack expression in the same region.
